# Prebiotic organic compounds in samples of asteroid Bennu indicate heterogeneous aqueous alteration

**DOI:** 10.1073/pnas.2512461122

**Published:** 2025-11-24

**Authors:** Angel Mojarro, José C. Aponte, Jason P. Dworkin, Jamie E. Elsila, Daniel P. Glavin, Harold C. Connolly, Dante S. Lauretta

**Affiliations:** ^a^National Aeronautics and Space Administration Postdoctoral Program, Oak Ridge Associated Universities, Oak Ridge, TN 37830; ^b^Solar System Exploration Division, National Aeronautics and Space Administration, Goddard Space Flight Center, Greenbelt, MD 20771; ^c^Lunar and Planetary Laboratory, University of Arizona, Tucson, AZ 85721; ^d^Department of Geology, School of Earth and Environment, Rowan University, Glassboro, NJ 08028; ^e^Department of Earth and Planetary Sciences, American Museum of Natural History, New York, NY 10024

**Keywords:** asteroid, sample return mission, soluble organic matter, insoluble organic matter, bennu

## Abstract

NASA’s OSIRIS-REx mission characterized the asteroid Bennu and delivered pristine samples of its regolith to Earth. Coordinated analyses of this primitive, carbonaceous material are elucidating the abiotic formation and inventory of prebiotic organic compounds in the early Solar System. Using pyrolysis and wet-chemistry techniques, we analyzed aggregate (unsorted particulate) material and three distinct stones that appear to correspond to different boulder types observed by the spacecraft. Results from the aggregate were consistent with previous work that detected the five canonical nucleobases and 14 of the 20 α-amino acids utilized by life to synthesize proteins. However, our analytical approach tentatively uncovered trace signals of a fifteenth α-amino acid, tryptophan, which has not been detected previously in extraterrestrial materials. Further, we found that the distributions of insoluble and soluble-derived organics differ between distinct stones, suggesting heterogeneous geologic processing within Bennu’s parent body. The distributions of alkylated polycyclic aromatic hydrocarbons resemble those in aqueously altered carbonaceous chondrites and are consistent with an abiotic origin through aqueous reactions. Our findings expand the evidence that prebiotic organic molecules can form within primitive accreting planetary bodies and could have been delivered via impacts to the early Earth and other Solar System bodies, potentially contributing to the origins of life.

## Significance

Samples of asteroid Bennu obtained by NASA’s OSIRIS-REx mission preserve a record of the chemical evolution of the early Solar System. The organic compounds detected in laboratory analyses of these samples include various building blocks of life, such as protein-synthesizing amino acids and the five nucleobases used in RNA and DNA. We found a previously undetected amino acid, tryptophan, which has not been observed previously in meteorites and returned samples. We also found that different types of rocks in the samples have distinct organic chemistries, indicating that the wet, alkaline environment in which they originated was host to heterogeneous aqueous reactions. Such asteroids may have seeded Earth and other bodies with the prebiotic inventory for the origin of life.

Asteroids and comets are among the most primitive objects known in our Solar System, predating the accretion of the terrestrial planets (1). They are composed of an amalgamation of components from the interstellar medium, our primordial solar nebula, and products of geologic processing formed within accreting planetary bodies (2). Organic-rich carbonaceous chondrite meteorites collected on Earth, originating from carbonaceous asteroids or extinct comets, contain various prebiotically relevant molecules including nucleobases (3) and amino acids (4–6). These compounds are the building blocks utilized by life on Earth to encode genetic information and mediate essential catalytic function (7). Observations and experimental studies indicate that many amino acids form via multiple reaction pathways mediated by the aqueous alteration of volatile-rich parent bodies (8) and that irradiation of interstellar ices can yield all canonical nucleobases utilized by life (9). Carbonaceous asteroids and comets therefore represent a significant extraterrestrial source which could have delivered volatiles and complex organics to planets and their satellites, contributing to the origins of life (10).

On 24 September 2023, samples collected from asteroid Bennu by the Origins, Spectral Interpretation, Resource Identification, and Security–Regolith Explorer (OSIRIS-REx) mission were delivered to Earth (11). These samples have been shown to preserve a record of the chemical evolution of the early Solar System (11–14). In contrast to meteorites which arrive without geological context and are contaminated by the terrestrial biosphere upon entry, samples returned by the OSIRIS-REx mission enable the study of pristine samples collected from a well-characterized body (15). One of the goals for this mission is to understand the abiotic formation and inventory of prebiotic organic compounds which may have been present in the early Solar System through the coordinated analysis of the Bennu samples (16). In particular, Bennu has been shown to contain organic matter with similar elemental and isotopic compositions, structures, and morphologies to those found in aqueously altered Ivuna-type (CI) and ungrouped (C-ung) carbonaceous chondrites, including insoluble organic matter (IOM) and soluble organic matter (SOM) (11, 12).

IOM is a major component of organic matter found in carbonaceous chondrite samples (>50% of the total organic carbon) that is insoluble in water, acids, and organic solvents (17). IOM is composed of a macromolecular network that resembles terrestrial kerogen, consisting of crosslinked polycyclic aromatic hydrocarbons (PAHs) and heterocyclic aromatic compounds (HACs) which are highly sensitive to modification by aqueous alteration (18). IOM may have originated from a common interstellar or nebular precursor overprinted by postaccretion parent-body processes (19). Variations in IOM composition between different petrographic types of carbonaceous chondrites may therefore reflect divergent geologic processing. Petrologic types 1 to 3 denote low-temperature aqueous alteration, with type 1 being the most aqueously altered and type 3 being the least (20). In contrast, SOM represents a smaller fraction (~1 to 3% total organic carbon) of freely extractable organics, including prebiotically relevant compounds (e.g., amino acids, nucleobases, hydrocarbons). SOM may be inherited from interstellar ices during accretion (21), synthesized in situ by chemical reactions within volatile-rich parent bodies mediated by aqueous alteration (2), and sourced from the conversion of labile IOM to SOM (22). Therefore, the molecular composition of IOM and SOM can be utilized to constrain parent-body alteration history and their chemical origins (4).

We conducted a targeted analysis of the twenty standard proteinogenic α-amino acids, β-alanine, γ-aminobutyric acid, the five canonical nucleobases, PAHs, and HACs to determine the prebiotic inventory across SOM and IOM reservoirs and derive their chemical origins within the context of Bennu’s parent-body alteration history. Specifically, the goal of this study is to elucidate relationships between the distribution of organic compounds across different particle types in the Bennu samples that may have experienced varying degrees and/or episodes of geological processing within Bennu’s parent body (11, 14). We performed the direct microanalysis of solid samples via pyrolysis and wet-chemistry approaches coupled to a gas chromatography–triple quadrupole–mass spectrometer (GC-QqQ-MS). Pyrolysis generally works through the thermal extraction of a sample’s labile (e.g., pyrolyzable) and volatile organic component (23), whereas the wet-chemistry derivatization technique is a modified procedure initially developed for the Sample Analysis at Mars (SAM) instrument aboard the Curiosity rover (24). We analyzed a homogenized powder of an aggregate sample consisting of unsorted particles <0.5 cm in size (OREX-800107-0) and homogenized powders of fragments from three stones, respectively, representing three broad categories of visually distinguishable particles identified in the returned Bennu samples (11): angular (OREX-800055-3), hummocky (OREX-800088-3), and mottled (OREX-800023-2). The particle types likely correspond to the different boulder types identified during the OSIRIS-REx remote-sensing campaign (11, 25, 26), and petrographic analysis has shown that at least the former two are distinct lithologies (14). Alongside the Bennu samples, we analyzed homogenized powders of CI, Mighei-type (CM), Renazzo-type (CR), and C-ung carbonaceous chondrites to contextualize pyrolysis products across different chondrite groups and petrologic classifications, as well as blanks of fused silica powder taken through all workups to characterize laboratory contributions (Table 1 and *SI Appendix*, Table S1).

**Table 1.** Samples of Bennu discussed in the main text

Parent		Designation	Analysis	Mass (mg)
OREX-800107-0	OREX-800107-189	aggregate	*pyQQQ*	2.7
	OREX-800107-118		*One-pot*	3.1
	OREX-800107-120		*One-pot*	2.8
	OREX-800107-122		*One-pot*	2.6
	OREX-800107-123		*One-pot*	3.2
OREX-800055-3	OREX-800055-112	angular	*pyQQQ*	1.1
	OREX-800055-113		*One-pot*	1
OREX-800088-3	OREX-800088-107	hummocky	*pyQQQ*	0.5
	OREX-800088-108		*One-pot*	0.7
OREX-800023-2	OREX-800023-102	mottled	*pyQQQ*	1
	OREX-800023-103		*One-pot*	0.8

pyQQQ: Standard pyrolysis in simultaneous fullscan and multiple reaction monitoring (MRM).

One-pot: Bulk derivatization (silylation) of powders by MTBSTFA: DMF (4:1) analyzed by MRM.

The complete inventory of Bennu and carbonaceous chondrite samples analyzed in this study is given in *SI Appendix*, Table S1.

## Results and Discussion

### Thermal Degradation Products of IOM.

We conducted pyrolysis–gas chromatography–triple quadrupole–mass spectrometry (pyGC-QqQ-MS) analyses by simultaneously scanning for unknown (*m*/*z* 50 to 500) and targeting known compounds from bulk pyrolyzed samples via multiple reaction monitoring (MRM). This technique contains precursor ion → product ion transitions for free organic volatiles and IOM thermal degradation products liberated by flash heating to ~610 °C (*SI Appendix*, Table S2). Approximately ~30% of IOM represents a pyrolyzable or “labile” fraction, while the remaining “refractory” char residue is never observed (27). Furthermore, a significant component of pyrolysates constitutes an unresolved complex mixture (UCM) that cannot be characterized by conventional gas chromatography (28).

**Fig. 1.**
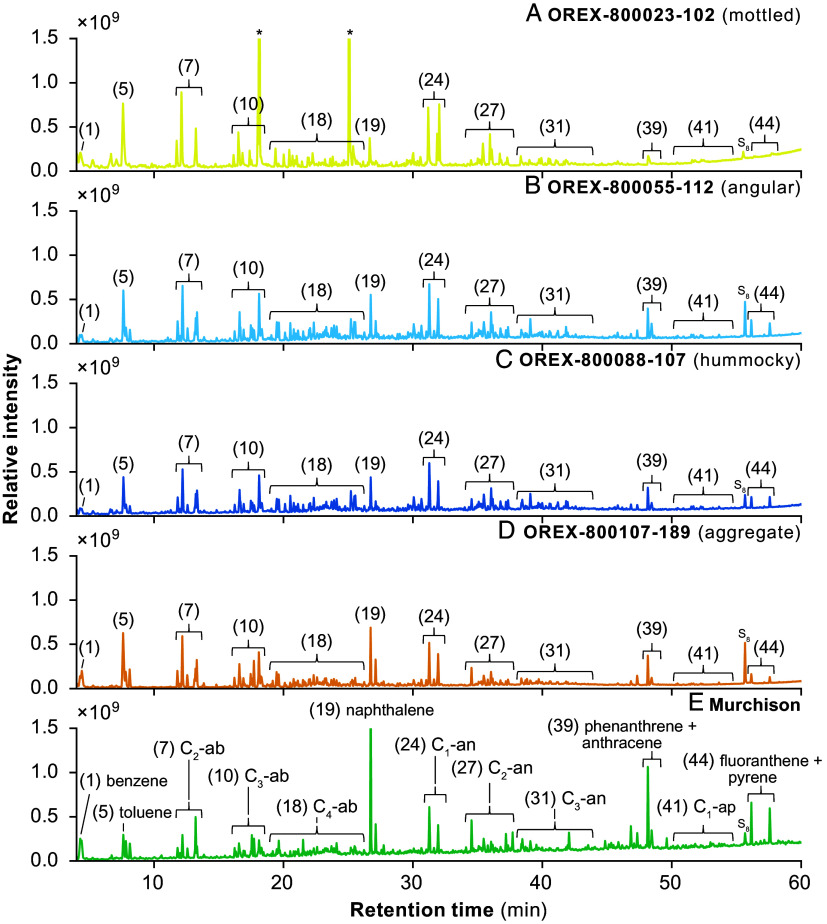
Free organic volatiles and IOM thermal degradation products liberated by flash heating to ~610 °C of Bennu samples. Shown are total ion chromatograms obtained by pyrolysis analysis of Bennu (*A*–*D*) (OREX-800023-102, OREX-800055-112, OREX-800088-107, OREX-800107-189) compared to (*E*) Murchison. Labels indicate major analytes and alkylation series detected: (1) benzene, (5) toluene, (7) C_2_-alkylbenzenes, (10) C_3_-alkylbenzenes, (18) C_4_-alkylbenzenes, (19) naphthalene, (24) C_1_-alkylnaphthalenes, (27) C_2_-alkylnaphthalenes, (31) C_3_-alkylnaphthalenes, (39) phenanthrene and anthracene, (41) C_1_-alkylphenanthrenes, (44) fluoranthene and pyrene. S_8_ denotes elemental sulfur, and asterisks (*) are siloxane peaks derived from a known laboratory contamination source. Fig. abbreviations are alkylbenzenes (ab), alkylnaphthalenes (an), alkylphenanthrenes (ap). The complete list of detected volatile and IOM-derived analytes is reported in *SI Appendix*, Table S2.

**Fig. 2.**
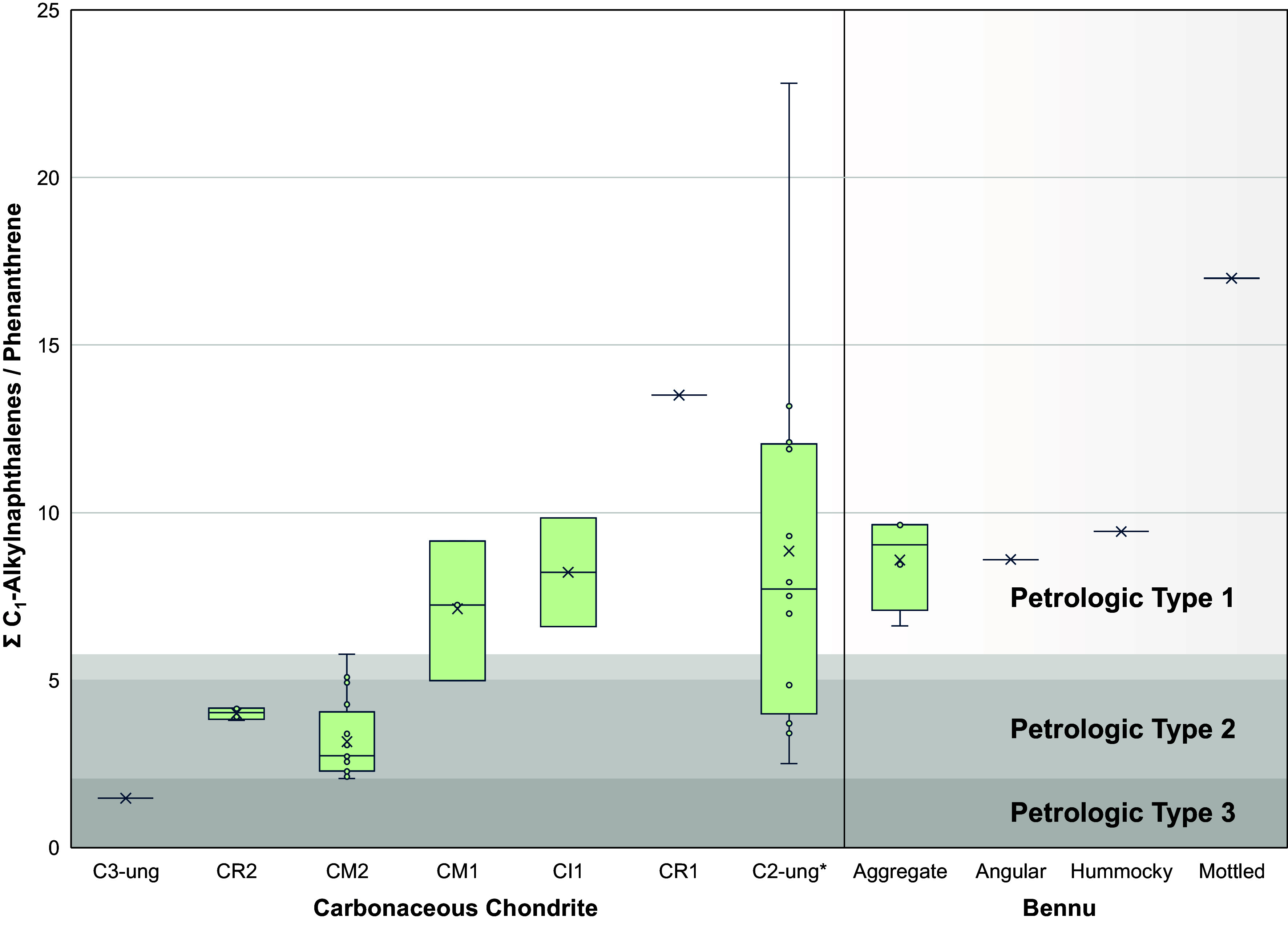
Relative abundances of free volatile and IOM-derived C_1_-alkylnapthalenes (*m*/*z* 142.1 → 141.1) versus phenanthrene (*m*/*z* 178.1 → 152.1) detected after flash pyrolysis (~610 °C) of Bennu and carbonaceous chondrite samples. The boxplot displays the interquartile range of ∑ C_1_-alkylnapthalenes/phenanthrene (∑ C_1_-Np/Ph) values; whiskers represent the minimum and maximum values. ∑ C_1_-Np/Ph is observed to record variable influence of aqueous alteration on the distribution and abundance of parent and alkylated PAHs. ∑ C_1_-Np/Ph values and sample details are given in *SI Appendix*, Table S1. *C2-ung includes various Tagish Lake meteorite lithologies which results in high variability; see *SI Appendix*, Fig. S1 and Table S1 for expanded ranges.

Pyrolysis of the aggregate (OREX-800107-189), angular (OREX-800055-112), hummocky (OREX-800088-107), and mottled (OREX-800023-102) samples primarily revealed highly alkylated benzenes, thiophenes, naphthalenes, benzothiophenes, phenanthrenes, and pyrenes characteristic of extensive aqueous alteration of the IOM within the Bennu parent body (29–31) (Fig. 1 and *SI Appendix*, Table S2). Most notably, angular, hummocky, and mottled samples displayed C_1_-alkylnaphthalene relative abundances (i.e., peak areas) greater than naphthalene (Fig. 1). Observations are consistent with naphthalene alkylation patterns displaying greater sensitivity to aqueous alteration relative to other PAHs (29). Comparison of ∑ C_1_-alkylnapthalenes/phenanthrene (∑ C_1_-Np/Ph) values among aqueously altered carbonaceous chondrites suggests that the Bennu samples (i.e., IOM) experienced similar alteration conditions as the CM1/CI1 and certain C2-ung meteorites (Fig. 2 and *SI Appendix*, Table S1). Though the exact mechanism for increased PAH alkylation remains uncertain, one possible explanation is due to condensation reactions between soluble and insoluble carbon reservoirs, resulting in the incorporation of smaller molecules to IOM and/or addition of alkyl groups to IOM moieties (32–34). The ∑ C_1_-Np/Ph ratio quantitatively compares the occurrence of alkylated and nonalkylated PAH thermal degradation products to reveal trends associated with increased aqueous alteration (29–31). Additional detections of PAHs include acenaphthylene, acenaphthene, phenalene, fluorene, anthracene, fluoranthene, and pyrene along with triphenylene, chrysene, and naphthacene (*SI Appendix*, Table S2).

Bennu chromatograms contain UCM characterized by an elevated baseline or “hump” from which discrete analytes are identified, not dissimilar to other meteorites analyzed in this study (Fig. 1). We detected nitrogen- and sulfur-containing compounds pyridine, aniline, benzonitrile, benzothiazole, indole, and carbazole (*SI Appendix*, Table S2). Possible sources include thermal degradation reactions from ammonia, sulfides, and soluble species such as amino acids (18, 35). Coordinated analyses have accordingly shown abundant ammonia and nitrogen-rich organic matter from OREX-800031-0, a Bennu aggregate sample (12). We also detected the sulfur-containing compounds dimethyl disulfide (DMDS), dimethyl trisulfide (DMTS), thiophene, benzothiophene, thienothiophenes, bithiophene, and dibenzothiophene, as well as elemental sulfur (S_8_) (*SI Appendix*, Table S2). DMDS and DMTS are likely derived from cleaved sulfide bridges crosslinking the IOM, while thiophenes partly originate from the cyclization of aliphatic sulfides (18). Previous work has shown that aqueous alteration of IOM increases the presence of macromolecularly bound heterocyclic organosulfur compounds, with CI1 carbonaceous chondrites (i.e., petrologic type 1) having the highest abundances relative to C2-ung, CM2, and CR2 (i.e., petrologic type 2) (36–38). We detected the oxygen-containing compounds benzaldehyde, phenol, benzofuran, dibenzofuran, benzophenone, and fluorenone (*SI Appendix*, Table S2), which could be derived by oxidation of organic matter by aqueous alteration (18, 27). Altogether, the organic composition of Bennu samples characterized by pyGC-QqQ-MS is consistent with molecular distributions observed in aqueously altered carbonaceous chondrite samples. Aggregate, angular, and hummocky samples effectively represent the average Bennu IOM signal consistent with CI1/CM1 chondrites (Fig. 2 and *SI Appendix*, Table S1). In contrast, organics from the mottled sample appear relatively more altered and comparable to Tagish Lake 5b as distinguished by the ∑ C_1_-Np/Ph ratio, though it is classified as a C2-ung (Fig. 2 and *SI Appendix*, Table S1).

### Derivatized SOM.

We performed one-pot reactions for the direct derivatization of SOM by heating the sample in *N*-(*tert*-butyldimethylsilyl)-*N*-methyltrifluoroacetamide:*N*,*N*-dimethylformamide (MTBSTFA:DMF 4:1 v/v) (39) (see *Methods* and *SI Appendix*). Derivatized samples were then analyzed by GC-QqQ-MS in MRM targeting the 20 standard proteinogenic α-amino acids, select nonproteinogenic amino acids, and a suite of N-heterocycles detected in carbonaceous chondrites and interstellar-ice laboratory analogs (9) (*SI Appendix*, Table S3).

**Fig. 3.**
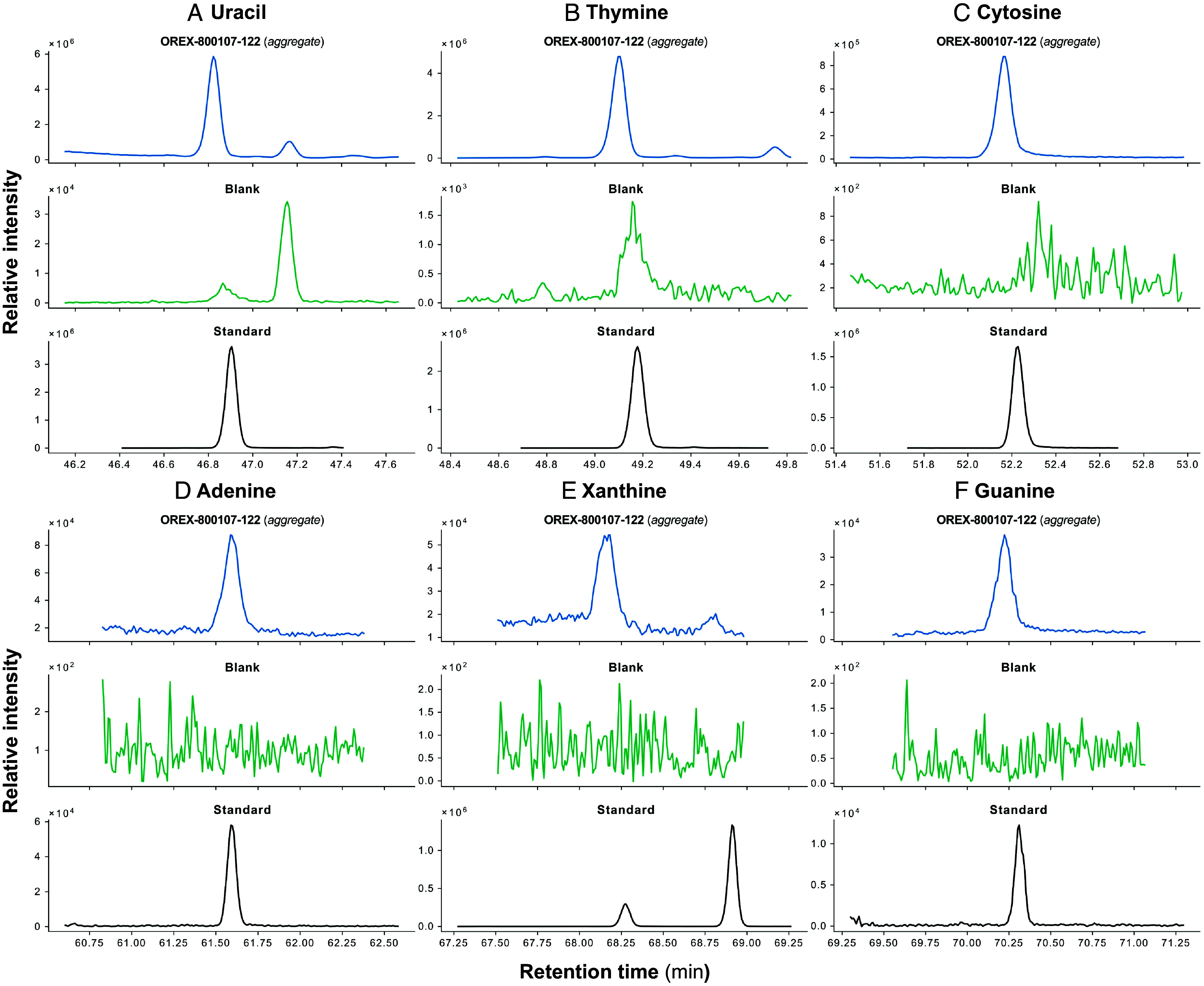
Nucleobases identified from one-pot derivatization (MTBSTFA:DMF) of a split (OREX-800107-122) of Bennu aggregate sample. Shown are multiple reaction monitoring (MRM) chromatograms of diagnostic product ions obtained by GC-QqQ-MS of silylated standards, fused silica (blank), and a Bennu sample (OREX-800107-122). (*A*) MRM chromatogram of uracil at *m*/*z* 283.1 → 147.1 corresponding to bisilylated (2tBDMS) uracil base peak. (*B*) MRM chromatogram of thymine at *m*/*z* 297.1 → 113 corresponding to bisilylated (2tBDMS) thymine base peak. (*C*) MRM chromatogram of cytosine at *m*/*z* 282.1 → 212.2 corresponding to bisilylated (2tBDMS) cytosine base peak. (*D*) MRM chromatogram of adenine at *m*/*z* 306.2 → 192.1 corresponding to bisilylated (2tBDMS) adenine base peak. (*E*) MRM chromatogram of xanthine at *m*/*z* 437.2 → 147.1 corresponding to trisilylated (3tBDMS) xanthine base peak. (*F*) MRM chromatogram of guanine at *m*/*z* 436.3 → 322.1 corresponding to trisilylated (3tBDMS) guanine base peak. N-heterocycles detected in the blank likely derive from background laboratory sources, solvents, or derivatization reagents used for sample processing and analysis.

We detected 15 proteinogenic α-amino acids and all five canonical nucleobases across quadruplicate splits (OREX-800107-118, OREX-800107-120, OREX-800107-122, OREX-800107-123) of the aggregate sample (Table 2 and Fig. 3 and *SI Appendix*, Fig. S2 and Table S3). Results from the splits of aggregate sample OREX-800107-0 in this study include amino acids and N-heterocycles (e.g., nucleobases) previously detected in hot-water and hydrochloric acid extracts of aggregate sample OREX-800031-0 (12). However, the previous study did not detect tryptophan (12), whereas here we found tentative trace signals of derivatized tryptophan across multiple replicate splits of OREX-800107-0 (*SI Appendix*, Figs. S3 and S4).

**Table 2.** Protein α-amino acids identified from one-pot derivatization (MTBSTFA:DMF) from Bennu aggregate and distinct stones

	OREX-800107-0^*^ (aggregate)	OREX-800055-113 (angular)	OREX-800088-108 (hummocky)	OREX-800023-103 (mottled)
Σ C_1_-MeNp/Ph:	6.6 to 9.6	8.6	9.4	17
Total α-AA Detected:	15/20	11/20	8/20	4/20
Glycine, *2tBDMS*	+	+	+	+
Alanine, *2tBDMS*	+	+	+	+
Serine, *3tBDMS*	+	+	−	−
Proline, *2tBDMS*	+	−	−	−
Valine, *2tBDMS*	+	+	+	+
Threonine, *3tBDMS*	+	+	+	−
Cysteine^†^, *4tBDMS*	−	−	−	−
Asparagine, 3*tBDMS*	+	−	−	−
Leucine, *2tBDMS*	+	+	+	+
Isoleucine, *2tBDMS*	+	+	−	−
Aspartic acid, *3tBDMS*	+	+	+	−
Glutamine, *3tBDMS*	−	−	−	−
Lysine, *3tBDMS*	−	−	−	−
Glutamic acid, *3tBDMS*	+	+	+	−
Methionine, *2tBDMS*	+	+	−	−
Histidine, 3*tBDMS*	−	−	−	−
Phenylalanine, *2tBDMS*	+	+	+	−
Arginine, 4*tBDMS*	−	−	−	−
Tyrosine, *3tBDMS*	+	−	−	−
Tryptophan, *3tBDMS*	+	−	−	−

^*^Analytes detected across all OREX-800107-0 replicate samples (splits).

^+^Analyte detected above background.

^−^Analyte not detected above background.

^†^Detected as a dimer of cysteine in standards.

The plus symbol (+) indicate detection of three MRM transitions above the derivatized fused silica (blank) and corroborated by standards. The minus symbol (–) indicates detection signal below the fused silica or below limits of detection. Detections from the Bennu aggregate sample (OREX-800107-0) span replicates of OREX-800107-118, OREX-800107-120, OREX-800107-122, and OREX-800107-123. Results from the angular (OREX-800055-113), hummocky (OREX-800088-108), and mottled (OREX-800023-103) stones are single measurements. The complete list of detections by MRM is reported in *SI Appendix*, Table S3.

Analytes detected are alanine, glycine, proline, valine, leucine, isoleucine, methionine, phenylalanine, threonine, serine, aspartic acid, glutamic acid, asparagine, tyrosine, tryptophan, uracil, thymine (5-methyluracil), cytosine, adenine, and guanine (*SI Appendix*, Table S3). We also detected nonproteinogenic amino acids, noncanonical nucleobases, and their isomers, including β-alanine, γ-aminobutyric acid, isocytosine, 6-methyluracil, 1-methyluracil, 2-imidazole carboxylic acid (2-ICA), 4-imidazole carboxylic acid (4-ICA), 5-methylcytosine, and xanthine (*SI Appendix*, Table S3). Identifications were above background levels and corroborated by analytical standards (Fig. 3 and *SI Appendix*, Fig. S2); they were additionally confirmed by three MRM transitions (*SI Appendix*, Fig. S2 and Table S3).

Detections from the individual stones were fewer relative to the aggregate sample: Eleven, eight, and four proteinogenic amino acids were respectively found in angular (OREX-800055-113), hummocky (OREX-800088-108), and mottled (OREX-800023-103) subsamples (Table 2 and *SI Appendix*, Table S3). Differences may be due to limited sample mass available from the stone fragments and heterogeneity at the low masses analyzed by this technique (Table 1). However, decreased amino acid diversity, as observed in the mottled sample, has been reported to correlate with increased aqueous alteration (40).

Prior analysis of Bennu aggregate sample OREX-800031-0 demonstrated racemic mixtures of chiral amino acids, confirming undetectable contributions from terrestrial contamination (12). Coanalysis of reagent blanks and one-pot derivatization reactions of fused silica powder yielded trace signals of alanine and glycine but not tryptophan (*SI Appendix*, Table S3), lending confidence to our detection. Also, it is unlikely that tryptophan and other reported compounds would be synthesized as a reaction byproduct, because MTBSTFA is a silylation reagent that adds a *tert*-butyldimethylsilyl (*t*BDMS) group to reactive polar groups (e.g., hydroxyl, carboxyl, amines, and amides) (41). Reactions occur at ∼85 °C, resulting in volatile and thermally stable derivatives; SOM present in bulk samples is presumably stabilized (derivatized) immediately prior to analysis by GC-QqQ-MS. Thermal degradation products of MTBSTFA from 50 to ~850 °C include alkylated benzenes, PAHs up to four rings, fluorinated compounds, and silylated water (42). These products are not expected to impact one-pot reactions occurring at low temperatures (∼85 °C).

### Implications for the Alteration History of Bennu’s Parent Body.

Bennu is a rubble pile, formed by reaccumulation of fragments originally produced by the catastrophic disruption of an older, larger parent body (43, 44). This event resulted in significant vertical mixing of heterogeneous and deep lithological layers that are now accessible to study at Bennu’s surface (26) and in returned samples (11).

The OSIRIS-REx remote-sensing campaign identified distinct boulder populations (25, 26) that likely correspond to the angular and hummocky stones (9). The angular stones are reminiscent of smooth, angular boulders on Bennu. They consist of 90 to 97 vol% fine-grained material that is >80 vol% phyllosilicates (serpentinite) with abundant magnetite and sulfides, indicating significant water–rock alteration (i.e., serpentinization) of a mafic protolith (11, 14). In contrast, the hummocky stones have a subrounded (cauliflower-like) morphology, like Bennu’s very dark and rough boulders, and have been identified as breccias, with some cemented by carbonate and magnetite veins (14). The hummocky matrix is phyllosilicate-rich and contains a variety of clasts, the majority of which are subangular to rounded, including fragments hypothesized to be derived from the angular lithology (14). If that is the case, the hummocky lithology is younger than the angular and later underwent lithification from subsequent water activity (14). The mottled stones are a less common component of the total returned sample, inferred to be related to a population of bright boulders displaying carbonate veins (45) and/or a bright variation of the angular lithology (25). The mottled stones are phyllosilicate-rich, mineralogically resembling the angular lithology, but with relatively high abundances of carbonates and Mg,Na-phosphates, suggesting extensive aqueous processing of an angular precursor (14).

Bennu samples analyzed in this study contain diverse organic compounds consistent with heterogeneous geological processing in the parent body. Pyrolysis of bulk samples revealed hydrocarbons, PAHs, and HACs, along with their highly alkylated homologs, analogous to aqueously altered meteorites (23). The aggregate, angular, and hummocky samples exhibit a range of alkylated to nonalkylated PAH ratios (Fig. 2 and *SI Appendix*, Fig. S1 and Table S1) resembling the organic inventories of type 1 carbonaceous chondrites, consistent with the pervasive aqueous alteration indicated by abundant phyllosilicates (46). In contrast, values from the mottled stone agree more closely with the most aqueously altered C2-ung carbonaceous chondrites (Fig. 2 and *SI Appendix*, Fig. S1 and Table S1)—in particular, the Tagish Lake meteorite, where IOM isolated from different stones displays a gradation of moderate to intense alteration (47).

Our wet-chemistry analyses of the Bennu samples reveal α-, β-, and γ-amino acids with putative origins from multiple aqueous reaction pathways (8) (discussed in the next section). In particular, among the individual stones, α-amino acid diversity decreased with increasing ∑ C_1_-Np/Ph values and petrologic evidence for multiple aqueous alteration events (14) (Fig. 2 and Table 2). The differences in aqueous alteration between the particle types relate to the number and timing of events experienced, rather than the linear progression in the extent of alteration that is traditionally interpreted as being recorded by different carbonaceous chondrites (4, 40). Our results suggest that organics derived from the angular lithology represent one major event, whereas those from hummocky and mottled particles represent more than one (Fig. 2 and Table 2). In the hummocky case, at least one additional event must have occurred to cement the aqueously altered clasts within the matrix; in the mottled case, a very late-stage fluid event is evidenced by the abundance of Mg,Na-rich phosphate, an evaporite mineral (11, 13, 14). Similarities between the angular and hummocky stones’ PAH ratios and α-amino acid diversity suggest a genetic link. An explanation for such a link could be that the phyllosilicate-rich mineralogy of the angular lithology and that of the phyllosilicate-rich clasts within the hummocky lithology were produced by the same event (14). Results from the aggregate sample are excluded from this comparison owing to probable detection biases in SOM-derived organics (i.e., amino acids) from the larger sample mass (up to 5 mg) relative to the limited mass of the stone fragments (less than 1 mg).

### Implications for Organic Matter Synthesis in Bennu’s Parent Body.

Remote (48) and laboratory analyses have shown that Bennu is a volatile-rich carbonaceous asteroid with abundant ammonia and nitrogen-rich organic matter (12). Significant D and ^15^N enrichments suggest that Bennu may be the remnant of a primitive icy body which experienced low-temperature aqueous alteration, likely mediated by ammonia brines (i.e., NH_3_⋅H_2_O) (11, 12). This interpretation is corroborated by widespread phyllosilicates, carbonate, magnetite, sulfides, and an evaporitic sequence of salts implying wet and alkaline conditions (11).

The diverse soluble organic compounds found in the three Bennu stones (Table 2 and *SI Appendix*, Table S3) are accordingly the products of a complex parent-body aqueous alteration history. Extraterrestrial amino acids can form through various reaction pathways that occur under specific conditions and utilize different organic feedstock molecules (4, 8, 49–51) (Table 3). Strecker synthesis is a well-known reaction mechanism whereby ammonia, cyanide, and a carbonyl (i.e., aldehyde or ketone) yield α-amino acids under aqueous conditions (52). However, this pathway primarily yields aliphatic α-amino acids and not aromatic species like tryptophan, tyrosine, and phenylalanine. Aromatic acids may be synthesized through Friedel–Crafts reactions catalyzed by water–rock interactions where aromatic hydrocarbon/heteroatom compounds are alkylated to produce the aromatic α-amino acids (53). Specifically, the proposed reaction mechanism is mediated by the serpentinization of a mafic protolith that generates hydrogen gas (H_2_), which then reacts with carbon monoxide (CO) or dioxide (CO_2_) to form small aromatic and aliphatic organics via Fischer– Tropsch-type (FTT) synthesis (54). Phyllosilicates can then adsorb molecules onto negatively charged surfaces to effectively concentrate organics and mediate alkylation of aromatic molecules with an α-keto acid followed by amination (55).

**Table 3.** Reaction mechanisms for amino acids and nucleobases detected in this study

Reaction mechanism	Reactants / Conditions	Major products	Notes
Strecker synthesis	Ammonia (NH_3_), cyanide (CN–), carbonyl (aldehyde/ketone), aqueous conditions	Aliphatic α-amino acids	Does not typically yield aromatic amino acids
Friedel–crafts alkylation and amination	Aromatic hydrocarbons/heteroatoms, α-keto acids; aqueous conditions, catalyzed by phyllosilicates	Aromatic α-amino acids (tryptophan, tyrosine, phenylalanine)	Mediated by water–rock interactions (serpentinization) producing H_2_ and subsequent Fischer–Tropsch-type synthesis of aromatic precursors
Michael addition	Ammonia (NH_3_), α,β-unsaturated nitriles, aqueous alteration conditions	α- and β-amino acids	Linked with extensive aqueous alteration, degradation of amino acids generates nitriles
Lactam hydrolysis and decarboxylation	α-amino dicarboxylic acids, aqueous conditions	γ- and δ-amino acids	γ-aminobutyric acid (GABA) as example detected
Formose-like condensation reaction	Ammonia (NH_3_), aldehydes (e.g., formaldehyde), alkaline conditions	α-, β-, γ-amino acids, nucleobases	Produces amino acid isomers via N-oxalylglycine intermediate; also a potential source for nucleobases
Ice photochemistry (solar nebula origin)	Solar nebula ices exposed to UV radiation	Nucleobases	Could be inherited directly from solar nebula photochemical reactions

Michael addition of ammonia to α,β-unsaturated nitriles is another reaction pathway likely responsible for the formation of α- and β-amino acids (56) detected in Bennu samples (Table 2 and *SI Appendix*, Table S3). Michael addition has been associated with extensive aqueous alteration within parent bodies, because α,β-unsaturated nitriles can form through α-amino acid degradation, and elevated β-amino acids relative to α-amino acids have been measured from CI1 chondrites and Tagish Lake 5b (4, 10, 56). γ-Amino acids (and δ-amino acids not screened in this study) have been suggested to form by lactam hydrolysis and decarboxylation of α-amino dicarboxylic acids (57). β-Alanine and γ-aminobutyric acid were the only non-α-amino acids utilized to evaluate the presence of diverse reaction networks (*SI Appendix*, Table S3). Experimental studies have additionally shown that α-, β-, and γ-amino acids may form via a Formose-like condensation reaction, where ammonia and aldehydes produce an N-oxalylglycine intermediate that is subsequently oxidized to produce various amino acid isomers (8). Moreover, glycine detected from cometary material (5, 6) has been inferred to form via ultraviolet photolysis of interstellar ice analogs (58). Nucleobases detected in Bennu may have been inherited from the solar nebula as products of ice photochemistry (9) or could have been synthesized from Formose-like condensation reactions of ammonia and formaldehyde under alkaline conditions (59).

Thus, the diverse amino acids and nucleobases we detected could have been inherited and synthesized through distinct reaction mechanisms within Bennu’s parent body (Table 3), possibly during different episodes of aqueous alteration, as recorded by the petrology of the different particle types (11, 14). Petrologic evidence (11, 14) and organics analyses (12) point toward pervasive and long-lived water–rock alteration responsible for the synthesis of prebiotically relevant compounds. However, further work is required to constrain the nature of ancient chemical reaction networks. Ongoing work on compound- and position-specific isotopes will further interrogate source materials (e.g., nebular feedstocks) and how molecules were assembled by tracing the fate of carbon through reaction steps (49, 50).

### Implications for Origins of Life.

We detected 15 of the 20 standard α-amino acids utilized by life to synthesize proteins, as well as the five canonical nucleobases. We did not observe cysteine, glutamine, lysine, histidine, and arginine. However, potential prebiotic pathways may exist for their synthesis within asteroid parent-bodies and prebiotic terrestrial environments. Specifically, modern biological systems synthesize cysteine from serine through reactions involving hydrogen sulfide (H_2_S) (60); analogous prebiotic chemistry could feasibly occur on the early Earth given meteoritic serine input and potential sulfide sources. Lysine originates from aspartic acid via diaminopimelic acid intermediates, suggesting a possible analogous prebiotic pathway to aspartic acid (61). Similarly, glutamic acid readily undergoes amination to produce glutamine in ammonia-rich aqueous environments, and it is also a known biological precursor to arginine via ornithine and citrulline intermediates (62). Furthermore, detected imidazole derivatives such as 2-imidazole carboxylic acid (2-ICA) and 4-imidazole carboxylic acid (4-ICA) are directly relevant precursors or intermediates for histidine formation through reductive amination, decarboxylation, or side-chain elongation reactions (63).

Little is known about the environments that may have existed when life originated, because extensive geologic resurfacing and recycling has erased Earth’s prebiotic history (64). Laboratory work such as the Miller–Urey spark experiment, meant to simulate an early dense and reducing atmosphere, exhibited the plausible synthesis of various amino acids (65) and nucleobases (66). Carbon-rich asteroids such as Bennu contain a complex organic inventory that could have been an additional source of prebiotically relevant compounds delivered to the early Earth, Mars, and ocean worlds (i.e., Europa and Enceladus). While the analytical approach presented here cannot quantify enantiomeric excesses associated with terrestrial contamination (67), limited background contribution observed in blanks and the presence of nonbiological isomers suggest an extraterrestrial origin (*SI Appendix*, Table S3). Notably, the tentative trace detection of tryptophan from Bennu could indicate that a labile fraction of organic compounds in meteorites does not survive atmospheric entry and descent to Earth. Therefore, the results shown here imply that tryptophan is not an exclusive product of biological metabolisms and therefore caution is warranted in using tryptophan as a definitive signature of life elsewhere (53, 67). Additional targeted analyses of tryptophan using other techniques capable of measuring its enantiomeric and isotopic compositions are needed to firmly establish its origin in Bennu and possibly other astromaterials. Sample return missions from a variety of planetary bodies are accordingly crucial to enabling new discoveries and elucidating products of cosmochemistry.

## Materials and Methods

Analytical methods used in this study have been adapted from pyrolytic techniques developed for the Sample Analysis at Mars (SAM) aboard the Curiosity Rover at Gale Crater (24). Noteworthy modifications include the implementation of MRM for targeted detection of analytes and operations unhindered by power requirements using modern laboratory equipment.

### Samples Used in This Investigation.

Analyses of Bennu material were conducted in parallel on multiple splits from a homogenized aggregate powder and three stone fragments, which were also homogenized by grinding in a quartz mortar and pestle in an ISO 5 flow bench in an ISO 7 whiteroom (*SI Appendix*). All tools and the fused silica blank had been baked at 550 °C overnight to remove organic contamination. All analyses were conducted alongside samples of the Murchison meteorite (Field Museum) and fused silica powder to serve as positive and negative controls. Additional Bennu aggregate samples analyzed by pyGC-QqQ-MS in this study listed in *SI Appendix*, Table S1 were indistinguishable from the larger sample allocation (OREX-800107-0) and are therefore not discussed in the main text. Tagish Lake samples were provided by Conel M. O’D. Alexander (Carnegie Science). Antarctic meteorite samples were recovered by the Antarctic Search for Meteorites (ANSMET) program which has been funded by NSF and NASA. Antarctic meteorites are characterized and curated by the Department of Mineral Sciences of the Smithsonian Institution and Astromaterials Curation Office at NASA Johnson Space Center.

### Analytical Methods.

Free volatile and IOM-derived compounds from Bennu and carbonaceous chondrites were detected by pyGC-QqQ-MS operated in simultaneous full scan (*m*/*z* 50 to 500) and MRM targeting known S-, O-, and N-heterocycles and hydrocarbon compounds informed by pyrolysis of CR, CI, CM chondrites and analogous coal standards. MRM transitions listed in *SI Appendix*, Table S2 were determined and optimized using the ThermoFisher AutoSRM software package. One-pot derivatization reactions occurred by incubating 5 µL of MTBSTFA:DMF (4:1 v/v) per 1 mg of sample at 85 °C for 1.5 h and vortexing every 15 min to prevent settling. Samples were then centrifuged, and the supernatant was manually injected for GC-QqQ-MS analysis in MRM only to filter unknown ions. MRM transitions listed in *SI Appendix*, Table S3 were determined by silylation of standards and selecting ions for optimal sensitivity and specificity using AutoSRM. ∑ C_1_-Np/Ph Data used in Fig. 2 and *SI Appendix*, Fig. S1, and listed in *SI Appendix*, Table S1 were determined using peak areas of methylnaphthalene transition *m*/*z* 142.1 → 141.1 and phenanthrene transition *m*/*z* 178.1 → 152.1 automatically integrated by the Chromeleon 7.3.1 Quantitation toolset. Detailed method steps and instrument parameters are described in *SI Appendix*.

## Supplementary Material

Appendix 01 (PDF)

Dataset S01 (XLSX)

## Data Availability

GCMS Files data have been deposited in Astromat (https://doi.org/10.60707/dej0-2v41 (68); https://doi.org/10.60707/2yvk-hf56 (69); https://doi.org/10.60707/m27f-0n07 (70); https://doi.org/10.60707/dnxd-z744 (71); https://doi.org/10.60707/3acp-zs80 (72); https://doi.org/10.60707/ezzn-v930 (73); https://doi.org/10.60707/ea9a-5c98 (74); https://doi.org/10.60707/zwxp-5y25 (75); https://doi.org/10.60707/43vs-yp59 (76); https://doi.org/10.60707/1k00-p463 (77); https://doi.org/10.60707/w7ka-gv63 (78); https://doi.org/10.60707/sxy1-sq73 (79); https://doi.org/10.60707/74r7-6915 (80); https://doi.org/10.60707/kacg-nb16 (81). All study data are included in the article and/or supporting information.
