# Supplementary material for: Prebiotic organic compounds in samples of asteroid Bennu indicate heterogeneous aqueous alteration
Source: Proc Natl Acad Sci U S A. 2025 Nov 24;122(49):e2512461122. doi: 10.1073/pnas.2512461122 (PMC12704803; doi:10.1073/pnas.2512461122)

Supporting Information for

Prebiotic organic compounds in samples of asteroid Bennu indicate heterogeneous aqueous alteration

Authors: Angel Mojarro[1,2,*], José C. Aponte[2], Jason P. Dworkin[2], Jamie E. Elsila[2], Daniel P. Glavin[2], Harold C. Connolly Jr.[3,4,5], and Dante S. Lauretta[3].

Affiliations:

[1]NASA Postdoctoral Program, Oak Ridge Associated Universities, Oak Ridge, Tennessee, USA.

[2]Solar System Exploration Division, NASA Goddard Space Flight Center, Greenbelt, Maryland, USA.

[3]Lunar and Planetary Laboratory, University of Arizona, Tucson, Arizona, USA.

[4]Department of Geology, School of Earth and Environment, Rowan University, Glassboro, New Jersey, USA.

[5]Department of Earth and Planetary Science, American Museum of Natural History, New York, New York, USA.

*Corresponding author: Angel Mojarro

Email: angel.mojarro@nasa.gov

This PDF file includes:

Supporting text

Figures S1 to S7

Tables S1 to S5

SI References

Other supporting materials for this manuscript include the following:

Figures S8 to S29: Amino acid structures and mass spectra

Figures S30 to S36: Nucleobase structures and mass spectra

Materials and Methods

Samples used in this investigation and controls. Analyses were conducted in parallel on multiple splits from a homogenized aggregate powder and stones extracted from the OSIRIS-REx Touch-and-Go Sample Acquisition Mechanism (TAGSAM). Bennu samples are designated aggregate (parent: OREX-800107-0), angular (parent: OREX-800055-3), hummocky (parent: OREX-800088-3), and mottled (parent: OREX-800023-2) (1). An additional Bennu aggregate (parent: OREX-800128-0) was utilized to collect ∑ C_1-alkylnapthalenes / phenanthrene (∑ C_1-Np/Ph) values, reported in **Table S1**; this sample is not discussed in the main text because pyrolysis results were indistinguishable from the larger OREX-800107-0 analysis (~1 mg vs. 2.7 mg, respectively). All analyses were conducted alongside samples of the Murchison CM2 meteorite, select carbonaceous chondrite meteorites (**Table S1**), and witness fused silica powder (FS-120, HP Technical Ceramics, Sheffield, UK) to characterize laboratory contributions. The two aggregate samples (6425 mg, OREX-800031-0; 205 mg, OREX-800128-0) consist of fine (<100 µm), intermediate (100–500 µm), and few larger particles up to 5 mm of mixed lithologies. Three stones, respectively representing two lithologies and one candidate lithology, were individually removed from TAGSAM: angular, 49 mg (OREX-800055-3); hummocky, 38 mg (OREX-800088-3); and mottled, 137 mg (OREX-800023-2). All Bennu samples were sealed inside crimp-top glass vials with polytetrafluoroethylene (PFTE) stopper and aluminum crimp under N_2 atmosphere at the NASA Johnson Space Center (JSC) astromaterials curation facility and shipped within discrete Eagle stainless containers (also under N_2) to the NASA Goddard Space Flight Center (GSFC). Sample handling, crushing, and subsampling for distribution occurred at the Astrobiology Analytical Laboratory (AAL) cleanroom laboratory within a HEPA-filtered laminar flow hood at atmosphere alongside witness fused silica (FS-120, HP Technical Ceramics, Sheffield, UK), and a previously characterized homogenized sample of Murchison CM2 (2). The complete sample handling and distribution process is described in SI Reference (3). A 65 mg subsample (OREX-800107-113) was allocated for all pyrolysis experiments discussed in the main text, while approximately ~1 mg of each stone and ~2 mg of the second Bennu aggregate (OREX-800128-0) were allocated for pyrolysis and one-pot derivatization reactions (**Table S1**).

Sample preparation and derivatization. All glassware used to handle samples, and the pyrolysis tubes themselves were previously ashed at 550°C for ~16 h in air. Bennu aggregate samples (OREX-800107-0, OREX-800128-0), stones (OREX-800055-3, OREX-800088-3, OREX-800023-2), fused silica, and carbonaceous chondrites were prepared for pyrolysis by loading powders into quartz pyroprobe tubes (CDS Analytical, Catalog: 6201-3004) inside a HEPA-filtered laminar flow bench. Sample-containing tubes were then transferred into the pyroprobe reaction furnace for analysis described below.

One-pot derivatization reactions were conducted by loading sample into 2 mL total-recovery vials and adding 5 µL *N*-(*tert*-butyldimethylsilyl)-*N*-methyltrifluoroacetamide (MTBSTFA):*N*,*N*-dimethylformamide (4:1 v/v) solution (MTBSFTA from Sigma Aldrich, >97% purity; DMF from Sigma Aldrich, anhydrous, 99.8% purity) per 1 mg of sample. Vials containing sample and reagent were then sealed with a PTFE liner and screw-on cap. Vials were then placed inside a stainless-steel heating block at 85°C for 1.5 h and vortexed every 15 min. Once complete, samples were centrifuged for 5 minutes, and 2–

5 uL of the supernatant was drawn with a 5 uL glass syringe for manual injection into the gas chromatograph.

Analytical Measurements and Discussion

Pyrolysis and Gas Chromatography–Triple Quadrupole Mass Spectrometry (Py-GC-QqQ-MS / GC-QqQ-MS). Pyrolysis experiments were conducted on a CDS Analytical 6200 pyroprobe configured for manual loading and flash (10°C ms^{-1}) heating ramps under a continuous flow (35 mL min^{-1}) of ultrahigh purity (>99.9 %) helium. Samples were heated in the pyrolysis furnace from 50 to 600°C (pyroprobe actual ~610°C) and held for 20 s to thermally degrade insoluble organic matter (IOM) and extract the stable hydrocarbon fraction. The pyroprobe housing and valves were held at 300°C, and volatiles were transferred via a heated transfer line (300°C) directly into a Thermo Scientific TRACE 1600 gas chromatograph (GC) coupled to an Thermo Scientific 9610 triple quadrupole mass spectrometer (TSQ) system. The GC inlet temperature was held at 300°C and operated with a 10:1 split. One-pot derivatization experiment analyses were conducted by manually injecting sample directly into the GC inlet held at 300°C and operated with a 2:1 split.

The GC was fitted with an Rtx-5MS capillary column (30 m × 0.25 mm × 0.25 µm) with 5 m Integra-Guard column, He carrier flow at 1.5 mL min^{-1}, and MS transfer line set to 300°C. The GC oven was programmed with the following method: 40°C hold for 5 min, followed by a 3.5°C min^{-1} ramp to 300°C, then a final isothermal hold at 300°C for 8.5 min (~88 min total). The MS source was held at 300°C and was operated in electron impact (EI) mode at 70 eV in either simultaneous fullscan (m/z 50–500) and multiple reaction monitoring (MRM) or only MRM. Py-GC-QqQ-MS and GC-QqQ-MS operated in MRM mode works by using specific precursor-product reactions in which Q1 scans for a specific precursor ion, filtered ions enter a collision cell (q), and Q3 scans for its diagnostic product ion. This approach results in a massively significant increase in detection sensitivity compared to fullscan as the quadrupoles focus a limited set of ions versus cycling across a broad range (m/z 50–500) every n-seconds (4). MRM therefore allows the detection of organic compounds at low abundances, enables more accurate analyte quantification due to detection specificity, and may potentially inform the discovery of previously undetectable compounds.

Pyrolysis included MRM transitions targeting IOM-derived hydrocarbons and S, N, and O-containing heterocycles informed by prior pyrolysis experiments on CM, CR, and CI carbonaceous chondrites and coal standards (**Table S2**). One-pot reactions included MRM transitions targeting silylated protein amino acids and N-heterocycles previously identified in meteorites (5, 6) and interstellar ice analogs (7–9) (**Table S3**). Pyrolysis blanks (1 mg fused silica) preceded all pyrolysis experiments to control the cleanliness of the analytical setup and prevent potential cross contamination. Injection of blanks (5 µL MTBSTFA:DMF and 1 mg fused silica) preceded one-pot experiments to characterize persisting background contamination of standards.

Results were analyzed using Chromeleon 7.3.1 software. Compound identification was conducted via comparison with retention time, three MRM transitions of standards, mass fragmentation patterns of standards, and comparison to previously characterized analog materials (i.e., CR, CI, CM carbonaceous chondrites and analogous coal standards). ∑ C_1-Np/Ph Data (**Dataset S1**) used in **Figure 2, Figure S1,** and listed in

Table S1 was determined using peak areas of methylnaphthalene transition *m/z* 142.1 → 141.1 and phenanthrene transition *m/z* 178.1 → 152.1 automatically integrated by the Chromeleon 7.3.1 Quantitation toolset.

Thermal degradation products of tryptophan. Further investigation was conducted to corroborate the tentative detection of extraterrestrial tryptophan from Bennu. Pyrolysis of all OSIRIS-REx samples included MRM transitions diagnostic for tryptophan and its thermal degradation products (10, 11). Due to its low volatility and thermal instability, tryptophan will primarily decarboxylate and deaminate to indole, 3-methylindole, 3-ethylindole, 2,3-dimethylindole, 3-ethyl-4-methylindole, and several thermal rearrangement isomers. Tryptophan will additionally decarboxylate to indole-3-acetonitrile and tryptamine and cyclize into quinoline, norharmane, harmane, and carbazole (11). Bennu aggregate (split: OREX-800107-189), angular (split: OREX-800055-112), hummocky (split: OREX-800088-107), and mottled (split: OREX-800023-102) samples all revealed detections of indole, various alkylated indoles, quinoline, and carbazole (**Table S4**). In contrast, pyrolysis of Murchison and additional CI, CM2, and C2-ung meteorites yielded indole, 3-methylindole without additional isomers, quinoline, and carbazole (**Table S4, Figure S5–S7**). Results suggest the detection of *n*-methylindoles, 3-ethylindole, 2,3-dimethylindole, 3-ethyl-4-methylindole, and several unidentified alkylated indole isomers are unique to Bennu samples (**Figure S5–S7**).

Figures and Tables

Figure S1. Expanded relative abundances of free volatile and IOM-derived C_1-alkylnapthalenes (*m*/*z* 142.1 → 141.1) **versus phenanthrene** (*m*/*z* 178.1 → 152.1) **detected after flash pyrolysis** (~610°C) **of Bennu and carbonaceous chondrite samples**. The boxplot displays the interquartile range of ∑ C_1-alkylnapthalenes / phenanthrene (∑ C_1-Np/Ph) values, whiskers represent the minimum and maximum values, and *n* denotes the number of sample replicates per sample-type when mass availability permitted multiple analyses. **Dataset S1** (∑ C_1-Np/Ph Calculations) contains all values utilized in **Figure 2** and **Figure S1**. Excluding ungrouped C2s and the mottled sample due to high variability, one-way ANOVA testing (in Python using scipy.stats.f_oneway) of petrologic type 1 (CI1, CM1, Bennu), type 2 (CR2, CM2), and type 3 (C3ung) indicates there is a significant difference between group means (p-value: $3.17\text{x}10^{-12}$). ∑C_1-Np/Ph values listed in **Table S1** used in this statistical test are:

Type 1: 5.0, 9.2, 7.2, 6.6, 9.8, 13.5, 6.6, 8.6, 9.4, 8.5, 9.6, 9.6

Type 2: 3.9, 4.1, 4.2, 3.8, 2.2, 2.1, 3.1, 5.1, 3.4, 4.3, 2.3, 2.6, 3.1, 5.8, 2.8, 2.1, 4.9, 5.0, 2.2, 2.4, 2.4, 2.1, 2.7, 2.6, 4.4, 2.3, 3.1, 3.1

Type 3: 1.6

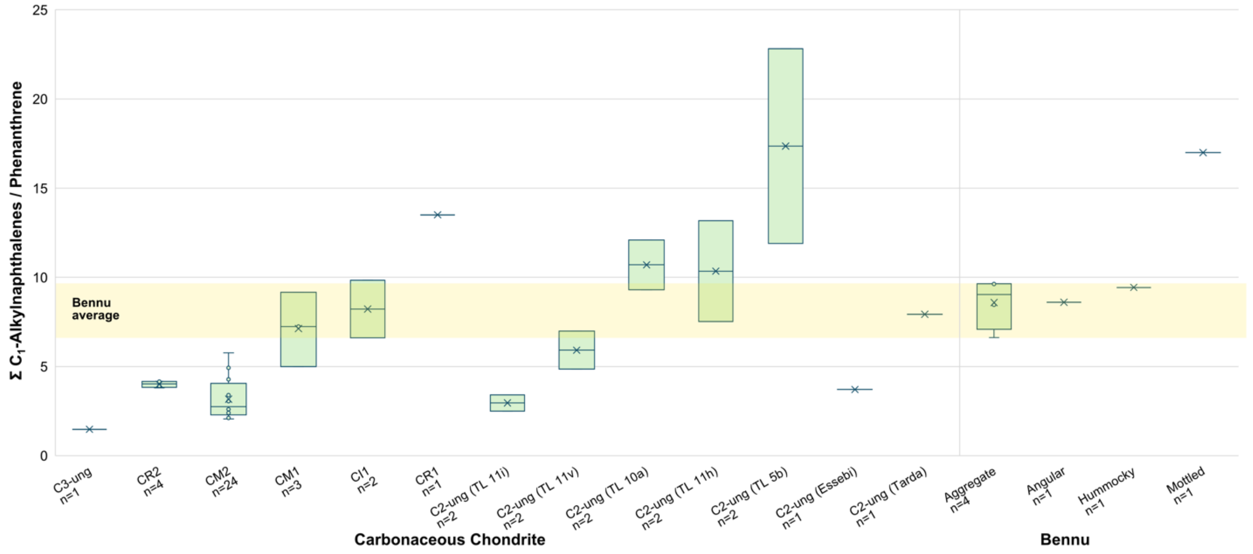

Figure S2. **Confirmation of nucleobases identified from one-pot derivatization** (MTBSTFA:DMF) **of Bennu aggregate** (split OREX-800107-122). Shown are three multiple reaction monitoring (MRM) chromatograms of diagnostic product ions utilized to corroborate the detection of silylated analytes by GC-QqQ-MS of the Bennu aggregate sample (OREX-800107-122). (**a**) MRM chromatogram of uracil quantitation peak *m*/*z* 283.1 → 147.1, confirmation peak 1 *m*/*z* 283.1 → 73, and confirmation peak 2 *m*/*z* 283.1 → 99.1. (**b**) MRM chromatogram of thymine quantitation peak *m*/*z* 297.1 → 113, confirmation peak 1 *m*/*z* 297.1 → 255.2, and confirmation peak 2 *m*/*z* 297.1 → 147.1. (**c**) MRM chromatogram of cytosine quantitation peak *m*/*z* 282.1 → 212.2, confirmation peak 1 *m*/*z* 282.2 → 213.2, and confirmation peak 2 *m*/*z* 282.1 → 170.1. (**d**) MRM chromatogram of adenine quantitation peak *m*/*z* 306.2 → 192.1, confirmation peak 1 *m*/*z* 306.2 → 193.1, and confirmation peak 2 *m*/*z* 192.1 → 165.1. (**e**) MRM chromatogram of xanthine quantitation peak *m*/*z* 437.2 → 147.1, confirmation peak 1 *m*/*z* 437.2 → 436.2, and confirmation peak 2 *m*/*z* 437.2 → 363.2. (**f**) MRM chromatogram of guanine quantitation peak *m*/*z* 436.3 → 322.1, confirmation peak 1 *m*/*z* 436.3 → 435.4, and confirmation peak 2 *m*/*z* 436.3 → 264.1. The complete list of MRM transitions utilized to detect and confirm amino acids and N-heterocycles is reported in **Table S3**.

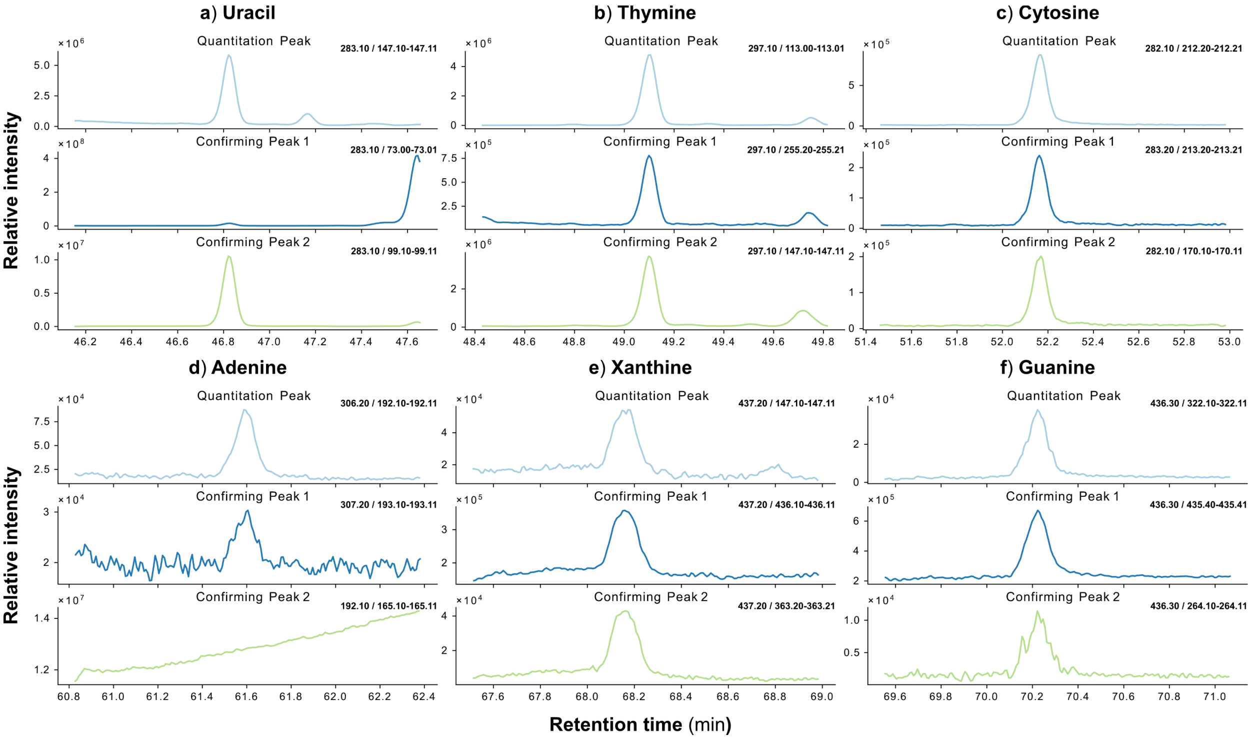

Figure S3. Tentative detection of derivatized tryptophan across splits of Bennu aggregate (OREX-8000107-0**).** Shown are multiple reaction monitoring (MRM) chromatograms of diagnostic product ions obtained by GC-QqQ-MS of trisilylated (3tBDMS) tryptophan (*m/z* 244.2 →188.1) across (**a**–**h**) replicate splits of Bennu aggregate sample (OREX-800107-118, OREX-800107-120, OREX-800107-122, OREX-800107-123), (**i**) fused silica (blank), (**j**) and a standard.

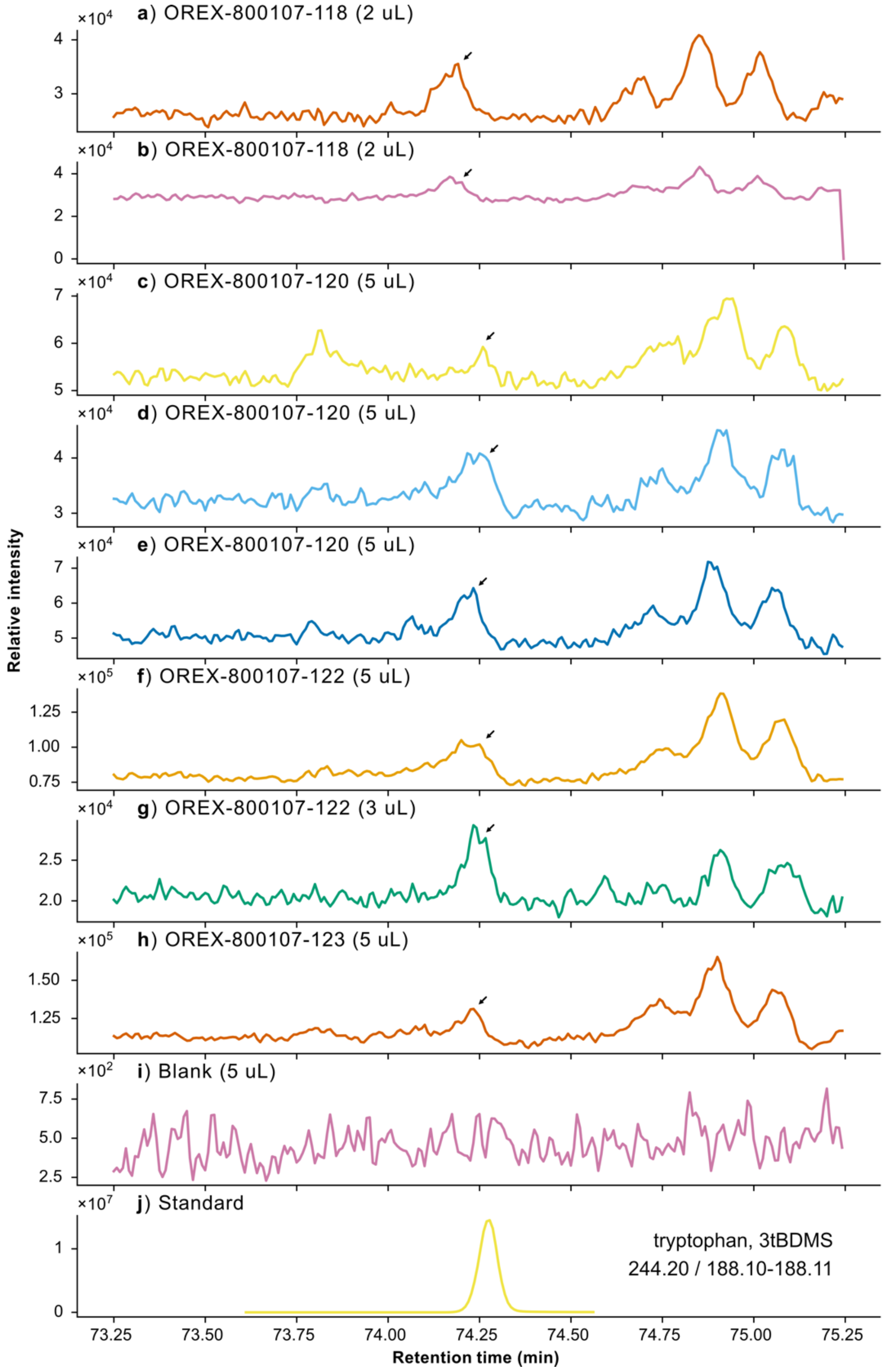

Figure S4. Tentative confirmation of derivatized tryptophan across splits of Bennu aggregate (OREX-8000107-0)**.** Shown are three multiple reaction monitoring (MRM) chromatograms of diagnostic product ions utilized to corroborate the detection of trisilylated (3tBDMS) tryptophan by GC-QqQ-MS from the (**a**–**h**) replicate splits of Bennu aggregate sample (OREX-800107-118, OREX-800107-120, OREX-800107-122, OREX-800107-123), (**i**) fused silica (blank), and (**j**) standard. The transitions are quantitation peak *m*/*z* 244.2 → 188.1, confirmation peak 1 *m*/*z* 245.2 → 189.2, and confirmation peak 2 *m*/*z* 244.2 → 73.1. Letters (**a**–**j**) correspond to the quantitation peaks shown in **Figure S3**. Identities of peaks to the left and right of the tentatively detected tryptophan are unknown.

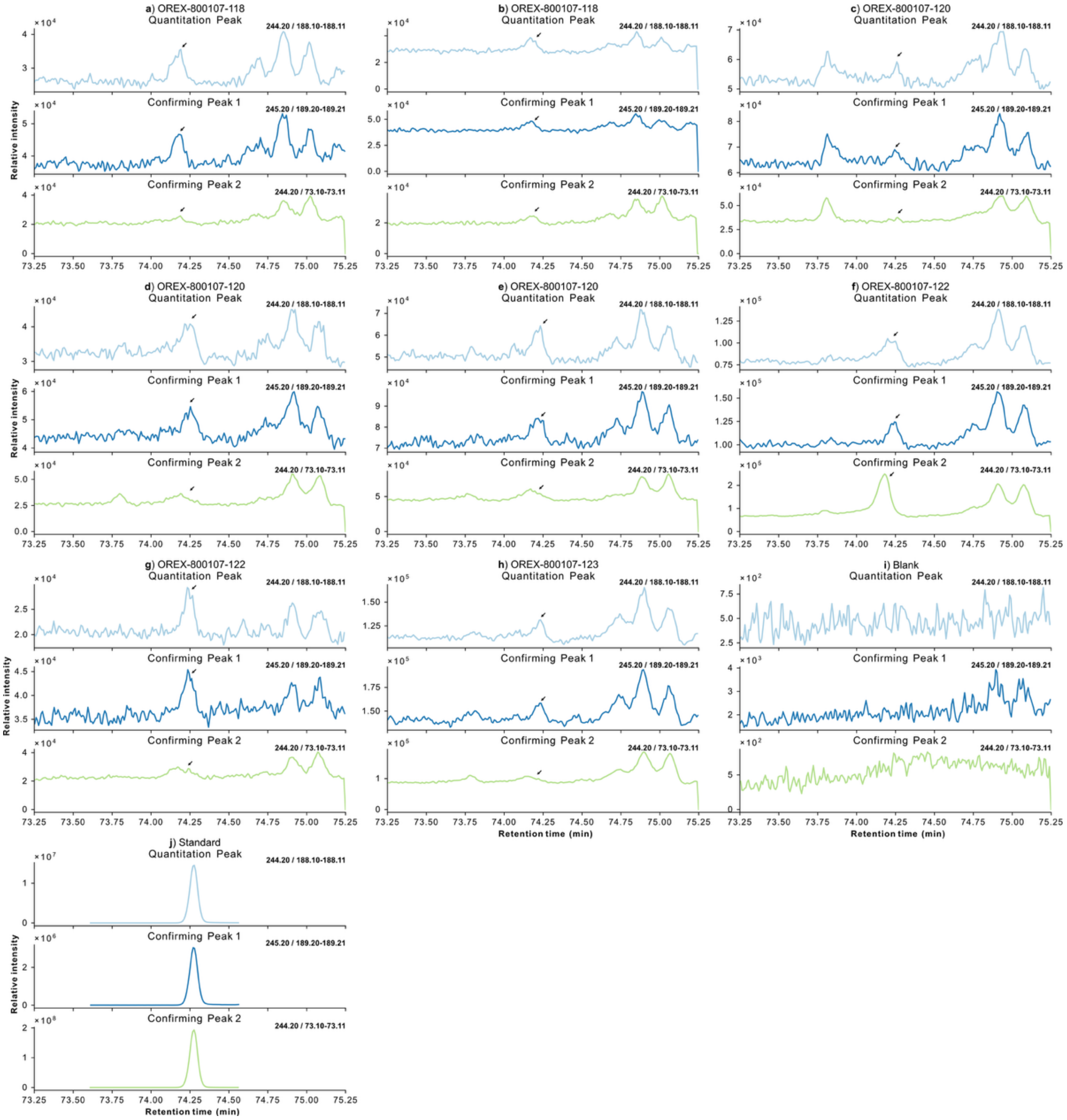

Figure S5. **Identification of indole in samples**. Shown are multiple reaction monitoring (MRM) chromatograms of diagnostic product ions of indole (*m*/*z* 117 → 89.1) detected by pyGC-QqQ-MS of (**a**–**d**) Bennu samples (OREX-800023-102, OREX-800055-112, OREX-800088-107, OREX-800107-189), (**e**) Murchison, (**f**) Tarda, (**g**) Tagish Lake 5b, (**h**) 0.1 mg of D/L-tryptophan powder (partially saturated detection), and (**i**) 1 mg of D/L-tryptophan powder (saturated detection).

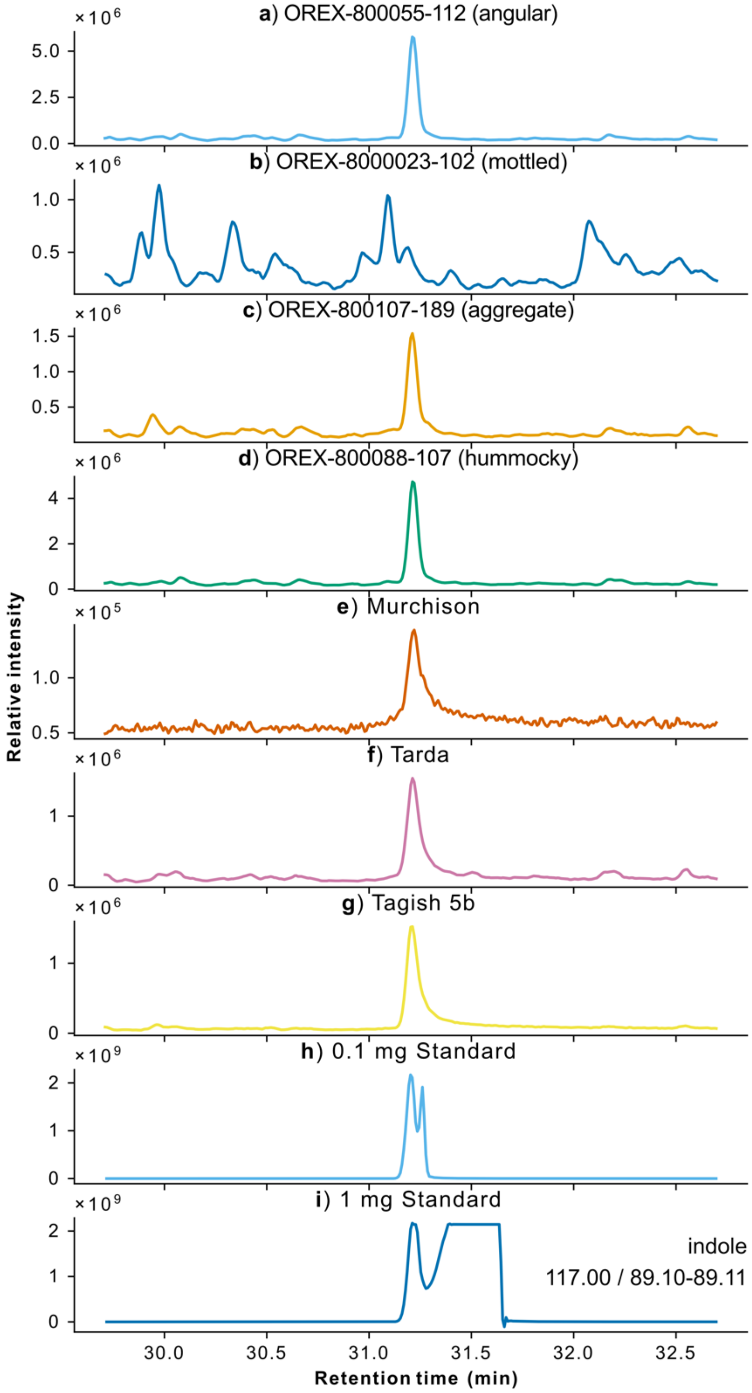

Figure S6. Identification of 3-methylindole and C_1-alkylindole isomers in samples. Shown are multiple reaction monitoring (MRM) chromatograms of diagnostic product ions of 3-methylinole and isomers (*m*/*z* 131.1 → 130.1) detected by pyGC-QqQ-MS of (**a**–**d**) Bennu samples (OREX-800023-102, OREX-800055-112, OREX-800088-107, OREX-800107-189), (**e**) Murchison, (**f**) Tarda, (**g**) Tagish Lake 5b, (**h**) 0.1 mg of D/L-tryptophan powder (partially saturated detection), and (**i**) 1 mg of D/L-tryptophan powder (saturated detection).

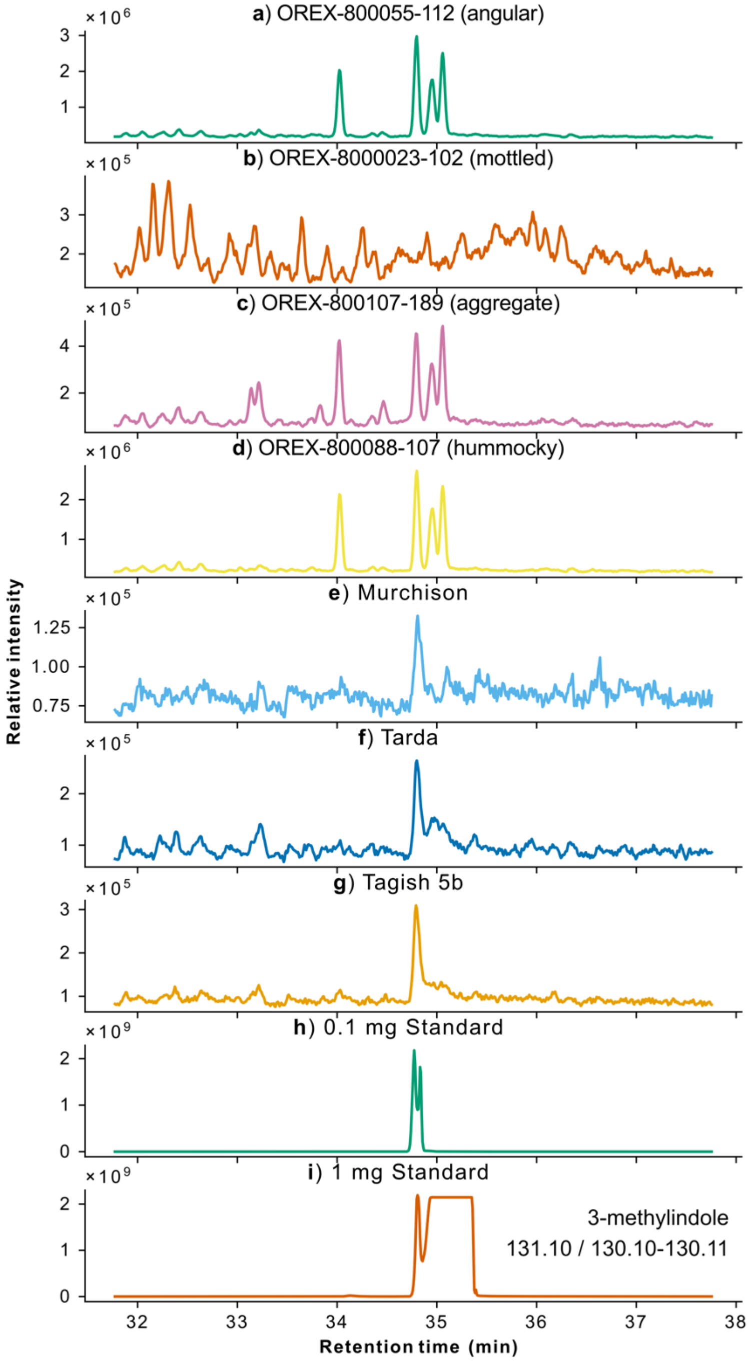

Figure S7. **Identification of 3-ethylindole and C_2-alkylindole isomers in samples**. Shown are multiple reaction monitoring (MRM) chromatograms of diagnostic product ions of 3-ethylindole (*m/z* 145.1 → 130) detected by pyGC-QqQ-MS of (**a**–**d**) Bennu samples (OREX-800023-102, OREX-800055-112, OREX-800088-107, OREX-800107-189), (**e**) Murchison, (**f**) Tarda, (**g**) Tagish Lake 5b, (**h**) 0.1 mg of D/L-tryptophan powder (partially saturated detection), and (**i**) 1 mg of D/L-tryptophan powder (saturated detection).

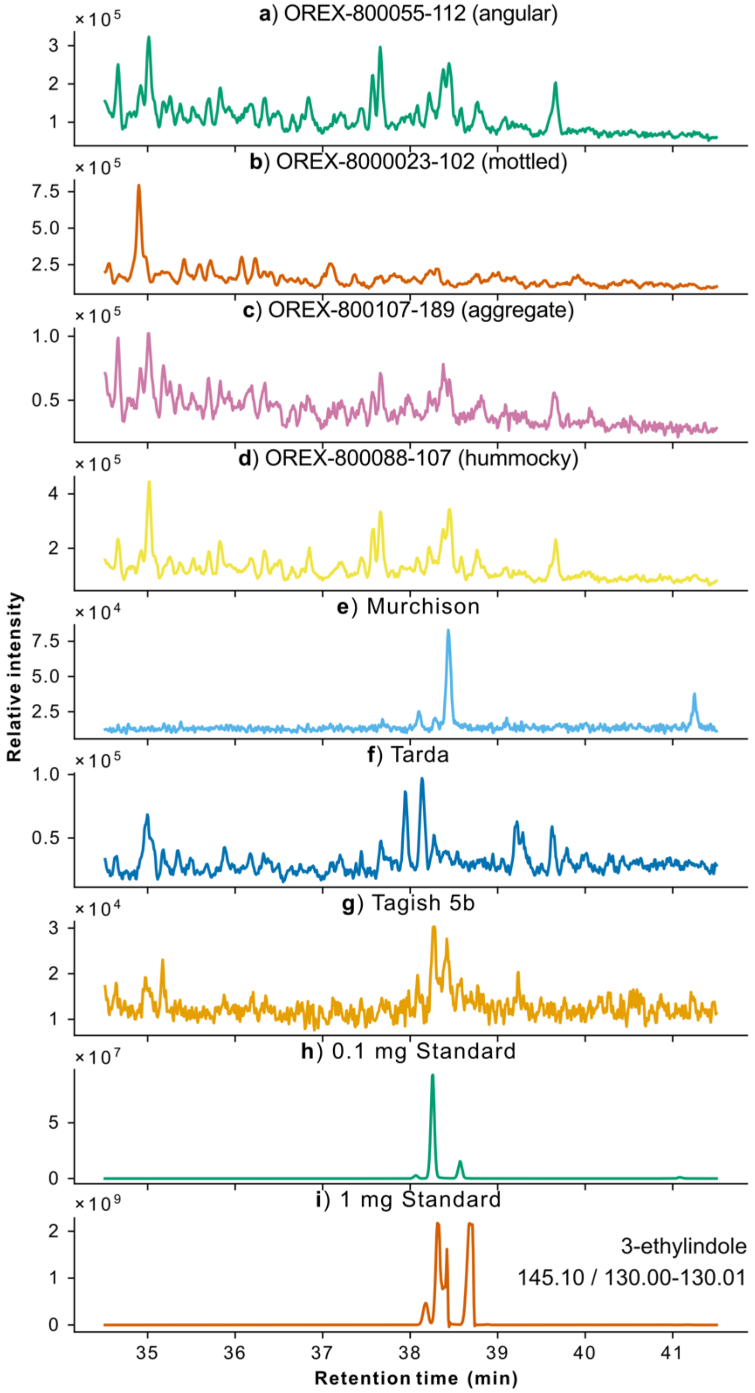

Table S1. All Bennu and carbonaceous chondrite samples analyzed, masses, and ∑ C_1-alkylnapthalenes (*m*/*z* 142.1 → 141.1) / phenanthrene (*m*/*z* 178.1 → 152.1) (∑ C_1-Np/Ph). Supplementary splits of Bennu aggregate samples (OREX-800107-114, OREX-800128-102, OREX-800128-103) were utilized to primarily measure additional ∑ C_1-Np/Ph and are not discussed in the main text.

Table S1: All Bennu and carbonaceous chondrite samples analyzed in this study.

**Parent**	**Split**	**Designation**	**Analysis**	**Mass** (mg)	**Σ C_1-Np/Ph**
		OSIRIS-REx			
OREX-800107-0	OREX-800107-114	aggregate	*pyQQQ*	1.1	9.6
	OREX-800107-189		*pyQQQ*	2.7	6.6
	OREX-800107-118		*One-pot*	3.1	-
	OREX-800107-120		*One-pot*	2.8	-
	OREX-800107-122		*One-pot*	2.6	-
	OREX-800107-123		*One-pot*	3.2	-
OREX-800128-0	OREX-800128-102	aggregate	*pyQQQ*	1.2	8.5
	OREX-800128-103		*pyQQQ*	1	9.6
OREX-800055-3	OREX-800055-112	angular	*pyQQQ*	1.1	8.6
	OREX-800055-113		*One-pot*	1	-
OREX-800088-3	OREX-800088-107	hummocky	*pyQQQ*	0.5	9.4
	OREX-800088-108		*One-pot*	0.7	-
OREX-800023-2	OREX-800023-102	mottled	*pyQQQ*	1	17
	OREX-800023-103		*One-pot*	0.8	-
	**Meteorite**	**Petrologic Type**	**Analysis**	**Mass** (mg)	**Σ C_1-Np/Ph**
		Carbonaceous Chondrites			
-	Orgueil	CI1	*pyQQQ*	1.4	6.6
-	Ivuna	CI1	*pyQQQ*	1	9.8
-	GRO 95577, 46	CR1	*pyQQQ*	1	13.5
-	SCO 06043,8	CM1	*pyQQQ*	1.3	5
-	MET 01070,34	CM1	*pyQQQ*	2	9.2
-	ALH 83100	CM1	*pyQQQ*	1	7.2
-	Murchison$^{n=11}$	CM2	*pyQQQ*	1 - 3	2.5 ± 0.3
-	Mighei$^{n=2}$	CM2	*pyQQQ*	1	2.3 ± 0.3
-	EET 96029	CM2	*pyQQQ*	1	3.4
-	LEW 85311	CM2	*pyQQQ*	1	4.3
-	LEW 90500$^{n=2}$	CM2	*pyQQQ*	1 -1.2	3.1
-	LON 94101$^{n=2}$	CM2	*pyQQQ*	1 - 1.8	5.4 ± 0.5
-	Murray	CM2	*pyQQQ*	2.6	2.1
-	LON 94102,19	CM2	*pyQQQ*	1	4.9
-	Jbilet Winselwan	CM2	*pyQQQ*	1	5
-	Winchcombe	CM2	*pyQQQ*	1.7	4.4
-	MIL 090657	CR2	*pyQQQ*	1.2	2.5
-	LAP 02342	CR2	*pyQQQ*	1	2.6
-	GRA 95229	CR2	*pyQQQ*	1	2.7
-	MIL 90001	CR2	*pyQQQ*	1.5	2.6
-	Tagish Lake 11i$^{n=2}$	C2-ung	*pyQQQ*	1	3 ± 0.6
-	Tagish Lake 11v$^{n=2}$	C2-ung	*pyQQQ*	1	5.9 ± 1.5
-	Tagish Lake 11h$^{n=2}$	C2-ung	*pyQQQ*	1	10.3 ± 4
-	Tagish Lake 10a$^{n=2}$	C2-ung	*pyQQQ*	1	10.7 ± 2
-	Tagish Lake 5b$^{n=2}$	C2-ung	*pyQQQ*	1	17.4 ± 7.7
-	Essebi	C2-ung	*pyQQQ*	1.4	3.7
-	Tarda	C2-ung	*pyQQQ*	3	7.9
-	Ningqian	C3-ung	*pyQQQ*	1.9	1.5

pyQQQ: Standard pyrolysis in simultaneous fullscan and multiple reaction monitoring (MRM).
One-pot: Bulk derivatization (silylation) of powders by MTBSTFA: DMF (4:1) analyzed by MRM.
Σ C_1-Np/Ph : Sum of C_1-alkylnaphthalenes (C_1-Np) / phenanthrene (Ph).
n: Number of sample replicates when mass availability permitted multiple analyses.

Table S2. Targeted free volatile and IOM-derived analytes with retention time and MRM transition utilized for identification from samples of Bennu.

Table S2: Targeted free volatile and IOM-derived analytes with retention time and MRM transition utilized for identification from samples of Bennu.

Number	Analyte	Retention Time (min)	Precursor Mass *m/z*	Product Mass *m/z*	Fused Silica	Murchison	OREX-800107-189 (aggregate)	OREX-800055-112 (angular)	OREX-800088-107 (hummocky)	OREX-800023-102 (mottled)
1	Benzene	4.3 ± 1	78.1	52.1	-	+	+	+	+	+
			78.1	63.1	-	+	+	+	+	+
			78.1	77.1	-	+	+	+	+	+
2	Thiophene	4.4 ± 1	84.1	45	-	+	+	+	+	+
			84.1	58	-	+	+	+	+	+
			84.1	69	-	+	+	+	+	+
3	Dimethyl disulfide	6.7 ± 1	94	61	-	+	+	+	+	+
			94	64	-	+	+	+	+	+
			94	79	-	+	+	+	+	+
4	Pyridine	6.8 ± 1.5	52.1	26.1	-	+	+	+	n.d.	+
			79.1	52.1	-	+	+	+	+	+
			79.1	77.1	-	n.d.	n.d.	n.d.	n.d.	n.d.
5	Toluene	7.6 ± 1	91.1	39.1	-	+	+	+	+	+
			91.1	65.1	-	+	+	+	+	+
			92.1	91.1	-	+	+	+	+	+
6	C_1-Alkylthiophenes	8 ± 1.5	97.1	45	-	+	+	+	+	+
			97.1	53.1	-	+	+	+	+	+
			97.1	69	-	+	+	+	+	+
7	C_2-Alkylbenzenes	12.5 ± 3	91.1	65.1	-	+	+	+	+	+
			105.1	77.1	-	+	+	+	+	+
			106.1	91.1	-	+	+	+	+	+
8	C_2-Alkylthiophenes	13 ± 3	111.1	77.1	-	+	+	+	+	+
			112.1	97	-	+	+	+	+	+
			112.1	111	-	+	+	+	+	+
9	Styrene	13.2 ± 1	78.1	52.1	-	+	+	+	+	+
			104.1	78.1	-	+	+	+	+	+
			104.1	103.1	-	+	+	+	+	+
10	C_3-Alkylbenzenes	18.5 ± 6	105.1	77.1	-	+	+	+	+	+
			105.1	103.1	-	+	+	+	+	+
			120.2	105.1	-	+	+	+	+	+
11	C_3-Alkylthiophenes	19 ± 8	125.1	97.1	-	+	+	+	+	+
			126.1	111	-	+	+	+	+	+
			126.1	125.1	-	+	+	+	+	+
12	Benzaldehyde	16.5 ± 1	77.1	51.1	-	+	+	+	+	+
			105.1	77.1	-	+	+	+	+	+
			106.1	105.1	-	+	+	+	+	+
13	Dimethyl trisulfide	17 ± 1	79	64	-	+	+	+	+	+
			126	79	-	+	+	+	+	+
			126	111.1	-	+	+	+	+	+
14	Aniline	17.4 ± 1	93.1	65.1	-	+	+	+	+	-
			93.1	66.1	-	+	+	+	+	-
			93.1	92.1	-	+	+	+	+	-
15	Phenol	17.6 ± 1	66.1	65.1	-	+	+	+	+	-
			94.1	39.1	-	+	+	+	+	-
			94.1	66.1	-	+	+	+	+	-
16	Benzonitrile	17.7 ± 1	76.1	50	-	+	+	+	+	+
			103.1	50	-	+	+	+	+	+
			103.1	76.1	-	+	+	+	+	+
17	Benzofuran	18.3 ± 1	90.1	63.1	-	+	+	+	+	+
			90.1	89.1	-	+	+	+	+	+
			118.1	117.1	-	+	+	+	+	+
18	C_4-Alkylbenzenes	23 ± 8	119.1	77.1	-	+	+	+	+	+
			119.1	91.1	-	+	+	+	+	+
			134.2	119.1	-	+	+	+	+	+
19	Naphthalene	26.8 ± 1	128.1	78.1	-	+	+	+	+	+
			128.1	102.1	-	+	+	+	+	+
			128.1	127.1	-	+	+	+	+	+
20	Benzo[c]thiophene	27.1 ± 1	134.1	89.1	-	+	+	+	+	+
			134.1	90.1	-	+	+	+	+	+
			134.1	108	-	+	+	+	+	+
21	Benzothiazole	28.5 ± 1	108	69	-	+	+	+	+	+
			135	91	-	+	+	+	+	+
			135	108	-	+	+	+	+	+
22	Thieno[n,n]thiophenes	28.5 ± 2	96.1	70	-	+	+	+	+	+
			96.1	95	-	+	+	+	+	+
			140	96	-	+	+	+	+	+
23	C_1-Alkylbenzo[b]thiophenes	31.5 ± 2	147.1	77.1	-	+	+	+	+	+
			147.1	103.1	-	+	+	+	+	+
			148.1	147	-	+	+	+	+	+
24	C_1-Alkylnaphthalenes	31.6 ± 1.5	141.1	115.1	-	+	+	+	+	+
			142.1	115.1	-	+	+	+	+	+
			142.1	141.1	-	+	+	+	+	+
25	Biphenyl	34.5 ± 1	76.1	63.1	-	+	+	+	+	+
			154.1	152.1	-	+	+	+	+	+
			154.1	153.1	-	+	+	+	+	+
26	Bithiophene	36 ± 3	121	77	-	+	+	+	+	+
			166	121	-	+	+	+	+	+
			166	134	-	+	+	+	+	+

27	C_2-Alkylnaphthalenes	36.1 ± 4	141.1	115.1	-	+	+	+	+	+
			156.1	115.1	-	+	+	+	+	+
			156.1	141.1	-	+	+	+	+	+
28	Acenaphthylene	37.2 ± 1	76.1	63.1	-	+	+	+	+	+
			152.1	126.1	-	+	+	+	+	+
			152.1	151.1	-	+	+	+	+	+
29	Acenaphthene	38.5 ± 1	153.1	152.1	-	+	+	+	+	+
			154.1	153.1	-	+	+	+	+	+
30	Dibenzofuran	39.5 ± 1	139.1	113	-	+	+	+	+	+
			168.1	139	-	+	+	+	+	+
			169.1	140	-	+	+	+	+	+
31	C_3-Alkylnaphthalenes	40.5 ± 5.5	155.2	128.1	-	+	+	+	+	+
			155.2	153.1	-	+	+	+	+	+
			170.2	155.1	-	+	+	+	+	+
32	Phenalene	41.2 ± 1.5	165.1	115	-	+	+	+	+	+
			165.1	139	-	+	+	+	+	+
			166.1	165.1	-	+	+	+	+	+
33	Fluorene	41.8 ± 1.5	165.1	95.1	-	+	+	+	+	+
			165.1	109.1	-	+	+	+	+	+
			166.1	165.1	-	+	+	+	+	+
34	Benzophenone	43.4 ± 1	105	77.1	-	+	+	+	+	+
			182.1	105	-	+	+	+	+	+
			182.1	181.1	-	+	+	+	+	+
35	C_4-Alkylnaphthalene	44 ± 6	169.1	153.1	-	+	+	+	+	+
			184.1	154.1	-	+	+	+	+	+
			184.1	169.1	-	+	+	+	+	+
36	C_5-Alkylnaphthalene	46 ± 7.5	183.1	153.1	-	-	+	+	+	+
			183.1	168.1	-	-	+	+	+	+
			198.1	183.2	-	-	+	+	+	+
37	Fluorenone	46.8 ± 1	152.1	126	-	+	+	+	+	+
			152.1	151.1	-	+	+	+	+	+
			180.1	152.1	-	+	+	+	+	+
38	Dibenzothiophene	47.3 ± 2	139.1	113	-	+	+	+	+	+
			184.1	139.1	-	+	+	+	+	+
			184.1	152.1	-	+	+	+	+	+
39	Phenanthrene + Anthracene	48.3 ± 2.5	178.1	152.1	-	+	+	+	+	+
			178.1	176.1	-	+	+	+	+	+
			178.1	177.1	-	+	+	+	+	+
40	Carbazole	49.8 ± 1	167.1	139.1	-	+	+	+	+	-
			167.1	140.1	-	+	+	+	+	-
			167.1	166.1	-	+	+	+	+	-
41	C_1-Alkylphenanthrenes	52 ± 4	191.1	165.1	-	+	+	+	+	+
			192.1	165.1	-	+	+	+	+	+
			192.1	191.1	-	+	+	+	+	+
42	Phenylnaphtalene	53.7 ± 1.5	204.1	189.1	-	+	+	+	+	+
			204.1	202.1	-	+	+	+	+	+
			204.1	203.1	-	+	+	+	+	+
43	C_2-Alkylphenanthrenes	55.2 ± 4	206.1	190.1	-	+	+	+	+	+
			206.1	191.1	-	+	+	+	+	+
			206.1	205.1	-	+	+	+	+	+
44	Fluoranthene + Pyrene	56.8 ± 2.5	202.1	200.1	-	+	+	+	+	+
			202.1	201.1	-	+	+	+	+	+
			101.1	100.1	-	+	+	+	+	+
45	C_3-Alkylphenanthrenes	58.5 ± 6	220.1	189.1	-	+	+	+	+	+
			220.1	190.1	-	+	+	+	+	+
			220.1	219.1	-	+	+	+	+	+
46	C_1-Alkylpyrenes/ Fluoranthenes	60 ± 3	216.1	215.1	-	+	+	+	+	+
			216.1	189.1	-	+	+	+	+	+
			216.1	214.1	-	+	+	+	+	+
47	C_2-Alkylpyrenes	60 ± 4	215.1	213.1	-	+	+	+	+	+
			230.1	215.1	-	+	+	+	+	+
			230.1	229.1	-	+	+	+	+	+
48	C_4-Alkylphenanthrenes	61 ± 5	219.1	189.1	-	-	-	-	-	-
			219.1	204.1	-	-	-	-	-	-
			234.1	219.1	-	-	-	-	-	-
49	C_3-Alkylpyrenes	65 ± 6	244.1	215.1	-	-	-	-	-	-
			244.1	228.1	-	-	-	-	-	-
			244.1	229.1	-	-	-	-	-	-
50	Terphenyl series	65 ± 5	230.1	215.1	-	+	+	+	+	+
			230.1	228	-	+	+	+	+	+
			230.1	229.1	-	+	+	+	+	+
51	Triphenylene/ Chrysene/ Naphthacene	65.1 ± 3	228.1	202.1	-	+	+	+	+	+
			228.1	226.1	-	+	+	+	+	+
			228.1	227.1	-	+	+	+	+	+

n.d.: Not determined due to co-eluting peaks.

Table S3. Targeted detection of silylated N-heterocycles and α-amino acids with retention time and MRM. Fused silica 1 is the analytical blank used for the Bennu aggregate sample (OREX-800107-0), while Fused silica 2 is the analytical blank used for the stones (OREX-800055-113, OREX-800088-108, OREX-800023-103). Select derivatized amino acid and nucleobase structures and mass spectra can be found in **Other supporting materials** (**Figures S8** - **S36**).

Table S3: Targeted detection of silylated N-heterocycles and amino acid with retention time and MRM transition utilized for identification from samples of Bennu.

Analyte	Retention Time (min)	Precursor Mass *m/z*	Product Mass *m/z*	Fused Silica 1	OREX-800107-0 (aggregate)	Fused Silica 2	OREX-800055-113 (angular)	OREX-800088-108 (hummocky)	OREX-800023-103 (mottled)
N-Heterocycles									
4-Pyrimidinone, 1-tBDMS	31.3 ± 1	99.1	45	n.d.	n.d.	n.d.	n.d.	n.d.	n.d.
		153.1	99	n.d.	n.d.	n.d.	n.d.	n.d.	n.d.
		154.1	100.1	n.d.	n.d.	n.d.	n.d.	n.d.	n.d.
Imidazole, 1-tBDMS	32.3 ± 1	125.1	98.1	n.d.	n.d.	n.d.	n.d.	n.d.	n.d.
		155.2	140.1	n.d.	n.d.	n.d.	n.d.	n.d.	n.d.
		182.2	126.1	n.d.	n.d.	n.d.	n.d.	n.d.	n.d.
1-Methylpyrazole-5-carboxylic acid, 1-tBDMS	36.8 ± 1	109.1	54.1	n.d.	n.d.	n.d.	n.d.	n.d.	n.d.
		139.1	59.1	n.d.	n.d.	n.d.	n.d.	n.d.	n.d.
		183.1	139.1	n.d.	n.d.	n.d.	n.d.	n.d.	n.d.
2-Ethyl-4-methylimidazole, 1-tBDMS	38.8 ± 1	167.1	109.1	-	n.d.	-	-	-	-
		168.1	113.1	-	n.d.	-	-	-	-
		224.2	168.2	-	n.d.	-	-	-	-
Isonicotinic acid, 1-tBDMS	38.9 ± 1	106	78.1	-	+	+	+	+	-
		180.1	106	-	+	+	+	+	-
		180.1	136.1	-	+	+	+	+	-
Isocytosine, 1-tBDMS	38.9 ± 1	168.1	74.1	-	n.d.	-	n.d.	n.d.	-
		168.1	99.1	-	n.d.	-	n.d.	n.d.	-
		168.1	126.1	-	n.d.	-	n.d.	n.d.	-
Nicotinic acid/ Picolinic acid, 1-tBDMS	39.8 ± 1	136.1	94.1	-	+	-	+	+	+
		180.1	106	-	+	-	+	+	+
		180.1	136.1	-	+	-	+	+	+
Picolinamide, 1-tBDMS	43.1 ± 1	179.1	75.1	-	+	+	+	+	+
Urea, 2-tBDMS	43.4 ± 1	147.1	131.1	+	+	+	+	+	+
		231.1	147	+	+	+	+	+	+
Nicotinamide, 1-tBDMS	44.9 ± 1	136.1	108.1	-	-	-	-	-	-
		179.1	105.1	-	-	-	-	-	-
		179.1	136.1	-	-	-	-	-	-
Isonicotinamide, 1-tBDMS	45.3 ± 1	136.1	108	-	-	-	+	+	+
		179.1	136.1	-	-	-	+	+	+
		180.1	137.1	-	-	-	+	+	+
2,4-Diaminopyrimidine, 1-tBDMS	45.6 ± 1	167.1	98.1	-	-	-	-	-	-
		167.1	125.1	-	-	-	-	-	-
		167.1	150.1	-	-	-	-	-	-
Purine, 1-tBDMS	46 ± 1	177.1	123.1	-	-	-	-	-	-
		178.1	136	-	-	-	-	-	-
		178.1	163.1	-	-	-	-	-	-
Uracil, 2-tBDMS	47 ± 1	283.1	73	+	+	+	+	+	+
		283.1	99.1	+	+	+	+	+	+
		283.1	147.1	+	+	+	+	+	+
6-Methyluracil, 2-tBDMS	48 ± 1	297.2	147.1	+	+	+	+	+	+
		298.2	148.1	+	+	+	+	+	+
		298.2	241.1	+	+	+	+	+	+
1-Methyluracil, 1-tBDMS	49.1 ± 1	100	72	-	n.d.	-	-	-	-
		183.1	72	-	n.d.	-	-	-	-
		183.1	100	-	n.d.	-	-	-	-
Thymine, 2-tBDMS	49.3 ± 1	297.1	113	+	+	+	+	+	+
		297.1	147.1	-	+	+	+	+	+
		297.1	255.2	-	+	+	+	+	+
Isocytosine, 2-tBDMS	49.9 ± 1	282.1	125.1	-	+	+	+	+	+
		282.1	171.1	-	+	+	+	+	+
		283.2	172.1	-	+	+	+	+	+
Imidazole-2-carboxylic acid, 2-tBDMS	50.3 ± 1	283.2	73.1	-	n.d.	+	n.d.	n.d.	n.d.
		283.2	239.2	-	+	+	+	+	+
Cytosine, 2-tBDMS	52.3 ± 1	282.1	170.1	-	+	+	+	+	-
		282.1	212.2	-	+	+	+	+	-
		283.2	213.2	-	+	+	+	+	-
5-Methylcytosine, 2-tBDMS	53 ± 1	296.2	112.1	-	+	-	-	-	-
		296.2	182.1	-	+	-	-	-	-
		296.2	226.2	-	+	-	-	-	-
2,4-Diaminopyrimidine, 2-tBDMS	54.4 ± 1	281.2	125.1	-	-	-	+	-	-
		281.2	170.1	-	+	-	+	-	-
		281.2	212.2	-	+	-	+	-	+
Imidazole-4-carboxylic acid, 2-tBDMS	58.3 ± 1	169.1	75.1	-	n.d.	-	n.d.	n.d.	n.d.
		169.1	125.1	-	+	-	+	+	+
		283.2	73.1	-	+	+	+	+	+
Adenine, 2-tBDMS	61.7 ± 1	192.1	165.1	-	+	-	-	-	-
		306.2	192.1	-	+	-	-	-	-
		307.2	193.1	-	+	-	-	-	-
2,6-Diaminopurine, 3-tBDMS	66.7 ± 1	321.2	73.1	-	-	-	-	-	-
		321.2	263.1	-	-	-	-	-	-
		321.2	305.2	-	-	-	-	-	-
Xanthine, 3-tBDMS	68.4 ± 1	437.2	147.1	-	+	-	-	-	-

		437.2	363.2	-	+	-	-	-	-
		437.2	436.1	-	+	-	-	-	-
Hypoxanthine, 2-tBDMS	69 ± 1.5	193.1	111	-	-	-	-	-	-
		307.2	193.1	-	-	-	-	-	-
		307.2	251.1	-	-	-	-	-	-
Guanine, 3-tBDMS	70.4 ± 1	436.3	264.1	-	+	-	-	-	-
		436.3	322.1	-	+	-	-	-	-
		436.3	435.4	-	+	-	-	-	-
2,6-Diaminopurine, 4-tBDMS	72.2 ± 1	435.3	263.1	-	+	-	-	-	-
		435.3	377.1	-	+	-	-	-	-
		435.3	419.2	-	+	-	-	-	-
				Amino Acids					
Alanine, 2-tBDMS	39.7 ± 1	158.2	73.1	+	+	+	+	+	+
		260.2	158.2	+	+	+	+	+	+
		260.2	232.2	+	+	+	+	+	+
Glycine, 2-tBDMS	40.5 ± 1	218.2	147.1	+	+	+	+	+	+
		246.1	147.1	+	+	+	+	+	+
		246.1	218.2	+	+	+	+	+	+
α-Aminoisobutyric acid (AIB), 2-tBDMS	41.6 ± 1	246.2	147.1	+	+	-	+	+	+
		274.2	147.1	+	+	-	+	+	+
		274.2	246.2	+	+	-	+	+	+
γ-Aminobutyric acid (GABA), 2-tBDMS	42 ± 1	246.2	147.1	+	+	+	+	+	+
		274.2	147.1	-	+	-	+	+	+
		274.2	246.2	-	+	-	+	+	+
β-Alanine, 2-tBDMS	43.1 ± 1	218.2	147.1	+	+	+	+	+	+
		260.2	117.1	-	+	-	+	+	+
		260.2	218.2	-	+	-	+	+	+
Valine, 2-tBDMS	43.6 ± 1	302.2	274.2	+	+	+	+	+	+
		260.2	147.1	+	+	+	+	+	+
		288.2	260.2	+	+	+	+	+	+
Leucine, 2-tBDMS	44.9 ± 1	274.2	147.1	+	+	+	+	+	+
		302.2	200.2	+	+	+	+	+	+
		302.2	274.2	+	+	+	+	+	+
Isoleucine, 2-tBDMS	45.9 ± 1	274.2	147.1	+	+	+	+	+	-
		302.2	147.1	+	+	+	+	-	-
		302.2	274.2	+	+	+	+	-	-
Proline, 2-tBDMS	47 ± 1	184.2	73.1	+	+	+	-	-	-
		258.2	147.1	+	+	+	-	-	-
		286.2	258.2	+	+	+	-	-	-
Pyroglutamic acid, 2-tBDMS	52.4 ± 1	147.1	131.1	-	+	+	-	-	-
		272.2	147.1	+	+	+	-	-	-
		300.1	272.2	+	+	+	-	-	-
Methionine, 2-tBDMS	52.9 ± 1	218.1	170.2	-	+	-	+	-	-
		292.2	147.1	-	+	-	+	-	-
		320.2	292.2	-	+	-	+	-	-
Serine, 3-tBDMS	53.4 ± 1	362.2	147.1	+	+	+	+	-	-
		390.2	230.2	+	+	+	+	-	-
		390.2	362.2	+	+	+	+	-	-
Threonine, 3-tBDMS	54.3 ± 1	303.2	148.1	-	+	-	+	+	-
		303.2	202.1	-	+	-	+	+	-
		303.2	287.2	-	+	-	+	+	-
Phenylalanine, 2-tBDMS	56.1 ± 1	234.2	178.1	+	+	+	+	+	-
		308.2	147.1	+	+	+	+	+	-
		336.2	308.2	+	+	+	+	+	-
Aspartic acid, 3-tBDMS	57.8 ± 1	302.2	147.1	-	+	+	+	+	-
		390.2	147.1	-	+	+	+	+	-
		390.2	346.3	-	+	+	+	+	-
Glutamic acid, 3-tBDMS	60.7 ± 1	272.2	147.1	-	+	+	+	+	-
		330.2	170.1	-	+	+	+	+	-
		432.3	272.2	-	+	+	+	+	-
Asparagine, 3-tBDMS	61.5 ± 1	302.2	147.1	-	+	-	-	-	-
		417.2	147.1	-	+	-	-	-	-
		417.2	400.2	-	+	-	-	-	-
Lysine, 3-tBDMS	63.2 ± 1	300.2	147.1	-	-	-	-	-	-
		300.2	168.1	-	-	-	-	-	-
		300.2	272.2	-	-	-	-	-	-
Glutamine, 3-tBDMS	64± 1.5	431.2	147.1	-	-	-	-	-	-
		431.2	271.2	-	-	-	-	-	-
		431.2	357.2	-	-	-	-	-	-
Arginine, 4-tBDMS	65.2 ± 2	442.3	199.2	-	-	-	-	-	-
		442.3	258.3	-	-	-	-	-	-
		442.3	283.2	-	-	-	-	-	-
Histidine, 3-tBDMS	68 ± 1	338.3	197.2	-	-	-	-	-	-
		440.3	280.1	-	-	-	-	-	-
		440.3	412.2	-	-	-	-	-	-
Tyrosine, 3-tBDMS	69 ± 1	302.2	147.1	-	+	-	-	-	-
		302.2	218.2	-	+	-	-	-	-
		302.2	245.1	-	+	-	-	-	-
Tryptophan, 2-tBDMS	69.8 ± 1	302.2	73.1	-	-	-	-	-	-
		302.2	147.1	-	-	-	-	-	-
		302.2	218.2	-	-	-	-	-	-
Tryptophan, 3-tBDMS	74.4 ± 2	244.2	73.1	-	+	-	-	-	-
		244.2	188.1	-	+	-	-	-	-
		245.2	189.2	-	+	-	-	-	-
Cysteine, 4-tBDMS	79.4 ± 1.5	348.2	106	-	-	-	-	-	-
		348.2	188.1	-	-	-	-	-	-
		348.2	302.2	-	-	-	-	-	-

n.d.: Not determined due to co-eluting peaks.

Table S4. Targeted detection of the thermal degradation products of tryptophan to ~610°C with retention times and MRM transitions.

Table S4: Targeted free volatile and IOM-derived analytes with retention time and MRM transition utilized for identification from samples of Bennu.

Analyte	Retention Time (min)	Precursor Mass *m/z*	Product Mass *m/z*	Fused Silica	D/L-Tryptophan	Murchison	OREX-800107-189 (aggregate)	OREX-800055-112 (angular)	OREX-800088-107 (hummocky)	OREX-800023-102 (mottled)
Quinoline	29 ± 1	129.1	102.1	-	+	+	+	+	+	+
		129.1	128.1	-	+	+	+	+	+	+
		102.1	102.1	-	+	+	+	+	+	+
Indole	31.2 ± 1.5	89	63	-	+	+	+	+	+	+
		90	89.1	-	+	+	+	+	+	+
		117	89.1	-	+	+	+	+	+	+
3-Methylindole	34.8 ± 3	130	77	-	+	+	+	+	+	-
		130	103.1	-	+	+	+	+	+	-
		131.1	130.1	-	+	+	+	+	+	-
3-Ethylindole/ 2,3-Dimethylindole	38 ± 3.5	130	77	-	+	-	+	+	+	-
		130	103.1	-	+	-	+	+	+	-
		145.1	130	-	+	-	+	+	+	-
3-Ethyl-4-methylindole	40.7 ± 4	144.1	115.1	-	+	-	+	+	+	-
		144.1	130.1	-	+	-	+	+	+	-
		159.1	144.1	-	+	-	+	+	+	-
Tryptamine	47 ± 3	130	103.1	-	+	-	-	-	-	-
		131.1	130.1	-	+	-	-	-	-	-
		160.1	131.1	-	+	-	-	-	-	-
Indole-3-acetonitrile	48.8 ± 1.5	155	101	-	+	-	-	-	-	-
		155	128.1	-	+	-	-	-	-	-
		156.1	155.1	-	+	-	-	-	-	-
Carbazole	49.8 ± 1	167.1	139.1	-	+	+	+	+	+	-
		167.1	140.1	-	+	+	+	+	+	-
		167.1	166.1	-	+	+	+	+	+	-
Indole-3-propionitrile	51.3 ± 1.5	170.1	130.1	-	+	-	-	-	-	-
		130	77	-	+	-	-	-	-	-
		130	103.1	-	+	-	-	-	-	-
Harman	53.4 ± 2	154.1	127.1	-	+	-	-	-	-	-
		182.1	154.1	-	+	-	-	-	-	-
		182.1	181.1	-	+	-	-	-	-	-
Norharman	53.9 ± 2	140.1	113	-	+	-	-	-	-	-
		168.1	114.1	-	+	-	-	-	-	-
		168.1	140.1	-	+	-	-	-	-	-
Tryptophan	59.6 ± 2.5	130	77	-	+	-	-	-	-	-
		130	103.1	-	+	-	-	-	-	-
		204.2	130.1	-	+	-	-	-	-	-

Table S5. List of the measurement data products from the Bennu samples analyzed in this study and corresponding DOIs available at https://astromat.org.

GCMS Data

DOI	Parent Sample	Split	Designation	Analysis	**Product Name** (Session ID)	Product Type
10.60707/dej0-2v41	OREX-800107-0	OREX-800107-114	aggregate	*pyQQQ*	20240701_GC-MS_GSFC_OREX-800107-114_1	GCMSCollection
10.60707/2yvk-hf56		OREX-800107-189		*pyQQQ*	20241029_GC-MS_GSFC_OREX-800107-189_1	GCMSCollection
10.60707/m27f-0n07		OREX-800107-118		*One-pot*	20240710_GC-MS_GSFC_OREX-800107-118_1	GCMSCollection
10.60707/dnxd-z744		OREX-800107-120		*One-pot*	20240708_GC-MS_GSFC_OREX-800107-120_1	GCMSCollection
10.60707/3acp-zs80		OREX-800107-122		*One-pot*	20240708_GC-MS_GSFC_OREX-800107-122_1	GCMSCollection
10.60707/ezzn-v930		OREX-800107-123		*One-pot*	20240709_GC-MS_GSFC_OREX-800107-123_1	GCMSCollection
10.60707/ea9a-5c98	OREX-800128-0	OREX-800128-102	aggregate	*pyQQQ*	20250122_GC-MS_GSFC_OREX-800128-102_1	GCMSCollection
10.60707/zwxp-5y25		OREX-800128-103		*pyQQQ*	20250122_GC-MS_GSFC_OREX-800128-103_1	GCMSCollection
10.60707/43vs-yp59	OREX-800055-3	OREX-800055-112	angular	*pyQQQ*	20241017_GC-MS_GSFC_OREX-800055-112_1	GCMSCollection
10.60707/1k00-p463		OREX-800055-113		*One-pot*	20241104_GC-MS_GSFC_OREX-800055-113_1	GCMSCollection
10.60707/w7ka-gv63	OREX-800088-3	OREX-800088-107	hummocky	*pyQQQ*	20241017_GC-MS_GSFC_OREX-800088-107_1	GCMSCollection
10.60707/sxy1-sq73		OREX-800088-108		*One-pot*	20241104_GC-MS_GSFC_OREX-800088-108_1	GCMSCollection
10.60707/74r7-6915	OREX-800023-2	OREX-800023-102	mottled	*pyQQQ*	20241205_GC-MS_GSFC_OREX-800023-102_1	GCMSCollection
10.60707/kacg-nb16		OREX-800023-103		*One-pot*	20241202_GC-MS_GSFC_OREX-800023-103_1	GCMSCollection

pyQQQ: Standard pyrolysis in simultaneous fullscan and multiple reaction monitoring (MRM).
One-pot: Bulk derivatization (silylation) of powders by MTBSTFA: DMF (4:1) analyzed by MRM.

SI References

1. D. S. Lauretta & H. C. Connolly Jr. *et al.*, Asteroid (101955) Bennu in the laboratory: Properties of the sample collected by OSIRIS-REx. *Meteorit. Planet. Sci.* **59**, 2453–2486 (2024).
2. D. P. Glavin *et al.*, Extraterrestrial amino acids and L-enantiomeric excesses in the CM2 carbonaceous chondrites Aguas Zarcas and Murchison. *Meteorit. Planet. Sci.* **56**, 148–173 (2021).
3. D. P. Glavin & J. P. Dworkin *et al.*, Abundant ammonia and nitrogen-rich soluble organic matter in samples from asteroid (101955) Bennu. *Nat. Astron.* **9**, 199–210 (2025). https://doi.org/10.1038/s41550-024-02472-9.
4. F. W. McLafferty, Tandem mass spectrometry. *Science* **214**, 280–287 (1981).
5. M. P. Callahan *et al.*, Carbonaceous meteorites contain a wide range of extraterrestrial nucleobases. *Proc. Natl. Acad. Sci. U S A* **108**, 13995–13998 (2011).
6. Y. Oba *et al.*, Identifying the wide diversity of extraterrestrial purine and pyrimidine nucleobases in carbonaceous meteorites. *Nat. Commun.* **13**, 2008 (2022).
7. C. K. Materese, M. Nuevo, S. A. Sandford, The formation of nucleobases from the ultraviolet photoirradiation of purine in simple astrophysical ice analogues. *Astrobiology* **17**, 761–770 (2017).
8. M. Nuevo, S. N. Milam, S. A. Sandford, Nucleobases and prebiotic molecules in organic residues produced from the ultraviolet photo-irradiation of pyrimidine in NH_3 and H_2O + NH_3 ices. *Astrobiology* **12**, 295–314 (2012).
9. Y. Oba, Y. Takano, H. Naraoka, N. Watanabe, A. Kouchi, Nucleobase synthesis in interstellar ices. *Nat. Commun.* **10**, 4413 (2019).
10. N. D. Danielson, L. B. Rogers, Determination of tryptophan in proteins by pyrolysis gas chromatography. *Anal. Chem.* **50**, 1680–1683 (1978).
11. R. K. Sharma, W. G. Chan, J. I. Seeman, M. R. Hajaligol, Formation of low molecular weight heterocycles and polycyclic aromatic compounds (PACs) in the pyrolysis of α-amino acids. *J. Anal. Appl. Pyrolysis* **66**, 97–121 (2003).

Other supporting materials

Amino acid structures and mass spectra. Shown are full scan (*m*/*z* 10 - 550) fragmentation patterns, selected precursor ions (bolded), and structures from derivatized standards of the 20 proteinogenic α-amino acids, β-alanine, and γ-aminobutyric acid. Retention times and ion transitions are listed in **Table S3**.

Figure S8. **Glycine, 2tBDMS**. Full scan (*m*/*z* 10 - 550) mass fragmentation of a bisilylated glycine derivative. The selected precursor ions are *m*/*z* **218.2** and **246.1**.

Molecular ion [M^+]: $C_{14}H_{33}NO_2Si_2$ (*m*/*z* 303.20)

[M^+] - *tert*-butyl (*t*B): $C_{10}H_{24}NO_2Si_2^{\bullet}$ (*m*/*z* 246.13)

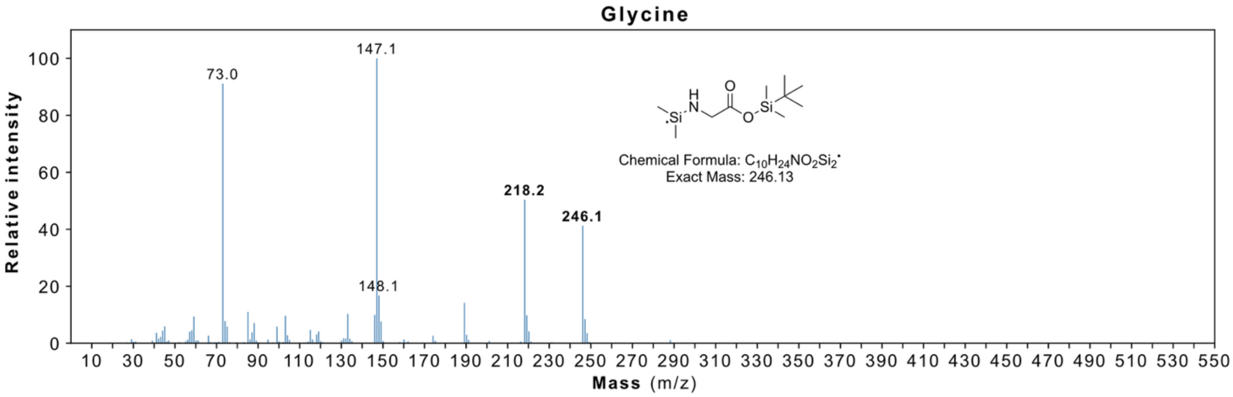

Figure S9. **Alanine, 2tBDMS**. Full scan (*m*/*z* 10 - 550) mass fragmentation of a bisilylated alanine derivative. The selected precursor ions are *m*/*z* **260.2** and **158.2**.

Molecular ion [M^+]: $C_{15}H_{35}NO_2Si_2$ (*m*/*z* 317.22)

[M^+] - *tert*-butyl (*t*B): $C_{11}H_{26}NO_2Si_2^{\bullet}$ (*m*/*z* 260.15)

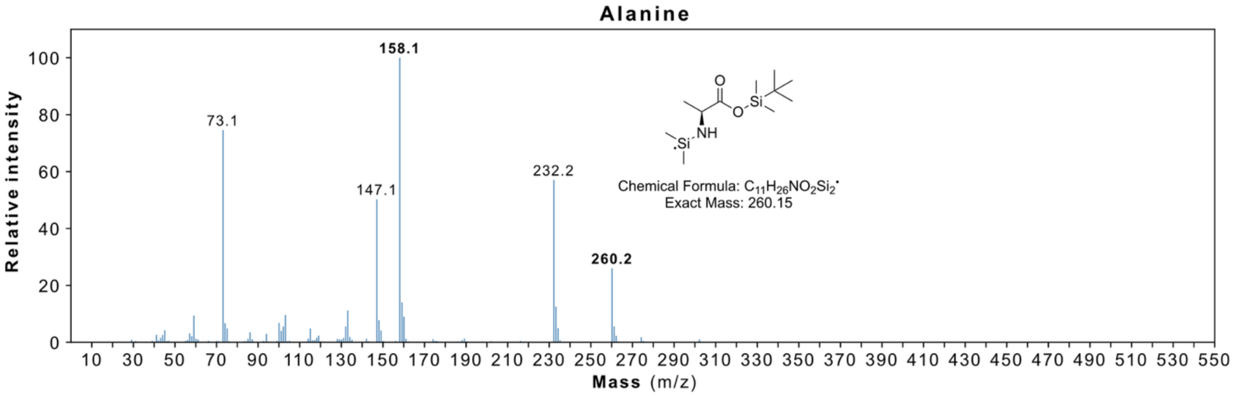

Figure S10. **β-Alanine, 2tBDMS**. Full scan (*m*/*z* 10 - 550) mass fragmentation of a bisilylated β-alanine derivative. The selected precursor ions are *m*/*z* **260.2** and **218.2**.

Molecular ion [M^+]: $C_{15}H_{35}NO_2Si_2$ (*m*/*z* 317.22)

[M^+] - *tert*-butyl (*t*B): $C_{11}H_{26}NO_2Si_2^{\bullet}$ (*m*/*z 260.15*)

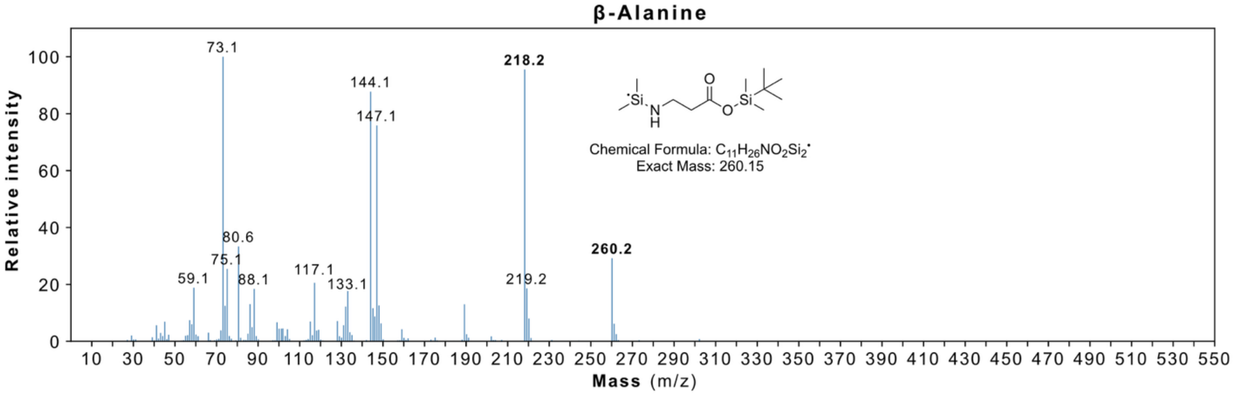

Figure S11. **y-Aminobutyric acid, 2tBDMS**. Full scan (*m*/*z* 10 - 550) mass fragmentation of a bisilylated y-aminobutyric acid derivative. The selected precursor ions are *m*/*z* **274.2** and **246.2**.

Molecular ion [M^+]: $C_{16}H_{37}NO_2Si_2$ (*m*/*z* 331.24)

[M^+] - *tert*-butyl (*t*B): $C_{12}H_{28}NO_2Si_2^{\bullet}$ (*m*/*z* 274.17)

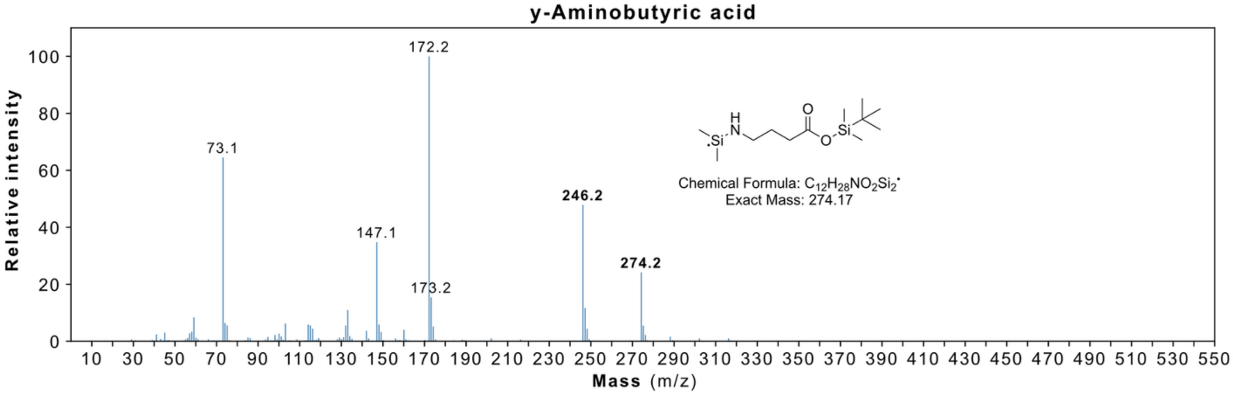

Figure S12. **Proline, 2tBDMS**. Full scan (*m*/*z* 10 - 550) mass fragmentation of a bisilylated proline derivative. The selected precursor ions are *m*/*z* **286.2**, **258.2**, and **184.2**.

Molecular ion [M$^+$]: $C_{17}H_{37}NO_2Si_2$ (*m*/*z* 343.24)

[M$^+$] - *tert*-butyl (*t*B):$C_{13}H_{28}NO_2Si_2^{\bullet}$ (*m*/*z* 286.17)

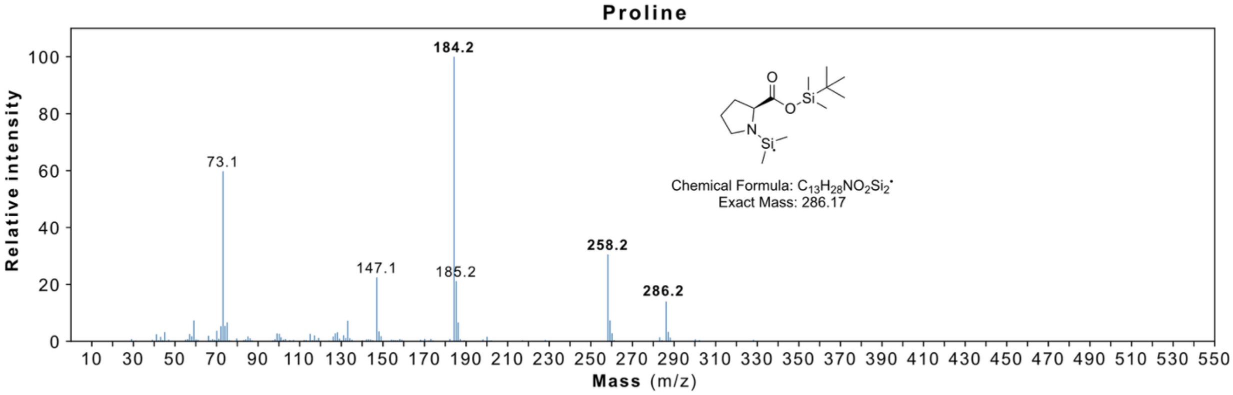

Figure S13. **Valine, 2tBDMS**. Full scan (*m*/*z* 10 - 550) mass fragmentation of a bisilylated valine derivative. The selected precursor ions are *m*/*z* **302.2**, **288.2**, and **260.2**.

Molecular ion [M$^+$]: $C_{17}H_{39}NO_2Si_2$ (*m*/*z* 345.25)

[M$^+$] - *tert*-butyl (*t*B): $C_{13}H_{30}NO_2Si_2^{\bullet}$ (*m*/*z* 288.18)

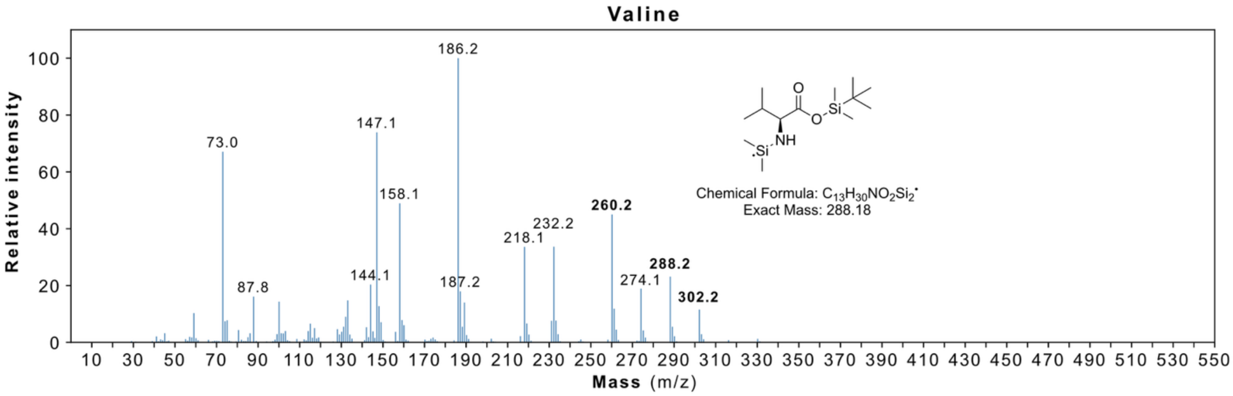

Figure S14. **Leucine, 2tBDMS**. Full scan (*m*/*z* 10 - 550) mass fragmentation of a bisilylated leucine derivative. The selected precursor ions are *m*/*z* **302.2** and **274.2**.

Molecular ion [M$^+$]: $C_{18}H_{41}NO_2Si_2$ (*m*/*z* 359.27)

[M$^+$] - *tert*-butyl (*t*B): $C_{14}H_{32}NO_2Si_2^{\bullet}$ (*m*/*z* 302.20)

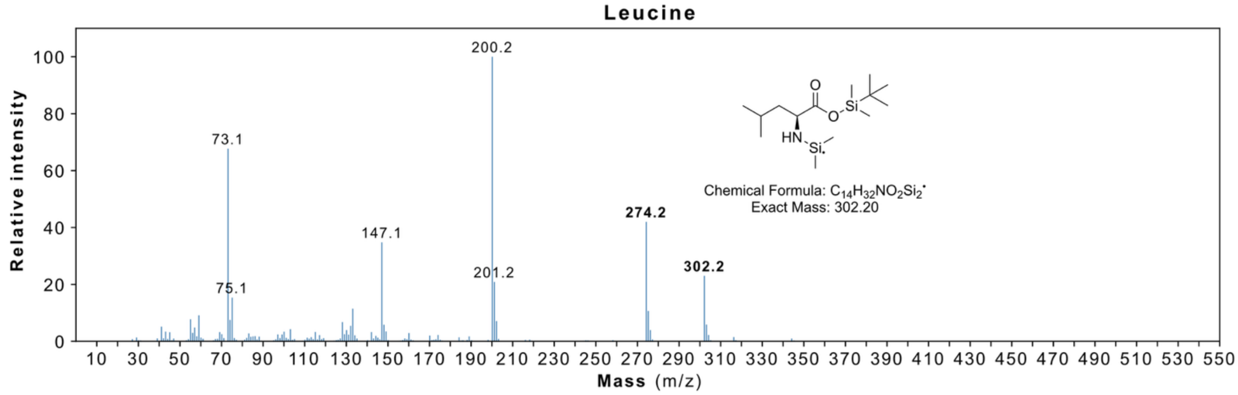

Figure S15. **Isoleucine 2tBDMS**. Full scan (*m*/*z* 10 - 550) mass fragmentation of a bisilylated isoleucine derivative. The selected precursor ions are *m*/*z* **302.2** and **274.2**.

Molecular ion [M$^+$]: $C_{18}H_{41}NO_2Si_2$ (*m*/*z* 359.27)

[M$^+$] - *tert*-butyl (*t*B): $C_{14}H_{32}NO_2Si_2^{\bullet}$ (*m*/*z* 302.20)

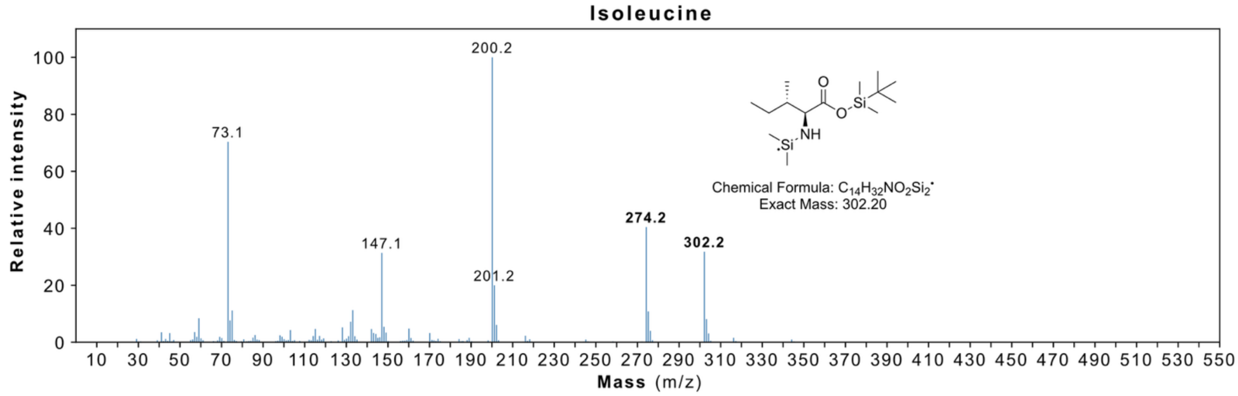

Figure S16. **Methionine, 2tBDMS**. Full scan (*m*/*z* 10 - 550) mass fragmentation of a bisilylated methionine derivative. The selected precursor ions are *m*/*z* **320.2**, **292.2**, and **218.1**.

Molecular ion [M+]: $C_{17}H_{39}NO_2SSi_2$ (*m*/*z* 377.22)

[M+] - *tert*-butyl (*t*B): $C_{13}H_{30}NO_2SSi_2^{\bullet}$ (*m*/*z* 320.15)

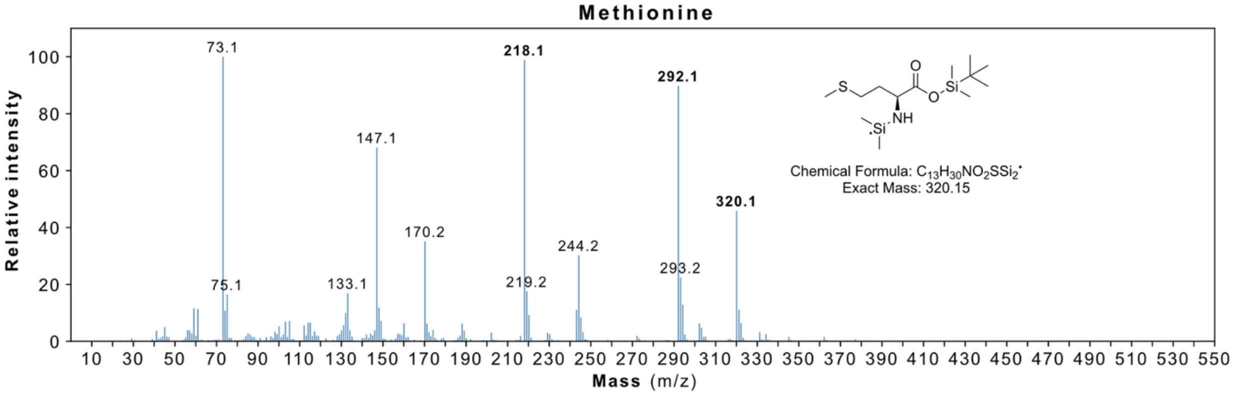

Figure S17. **Phenylalanine, 2tBDMS**. Full scan (*m*/*z* 10 - 550) mass fragmentation of a bisilylated phenylalanine derivative. The selected precursor ions are *m*/*z* **336.2**, **308.2**, and **234.2**.

Molecular ion [M+]: $C_{21}H_{39}NO_2Si_2$ (*m*/*z* 393.25)

[M+] - *tert*-butyl (*t*B): $C_{17}H_{30}NO_2Si_2^{\bullet}$ (*m*/*z* 336.18)

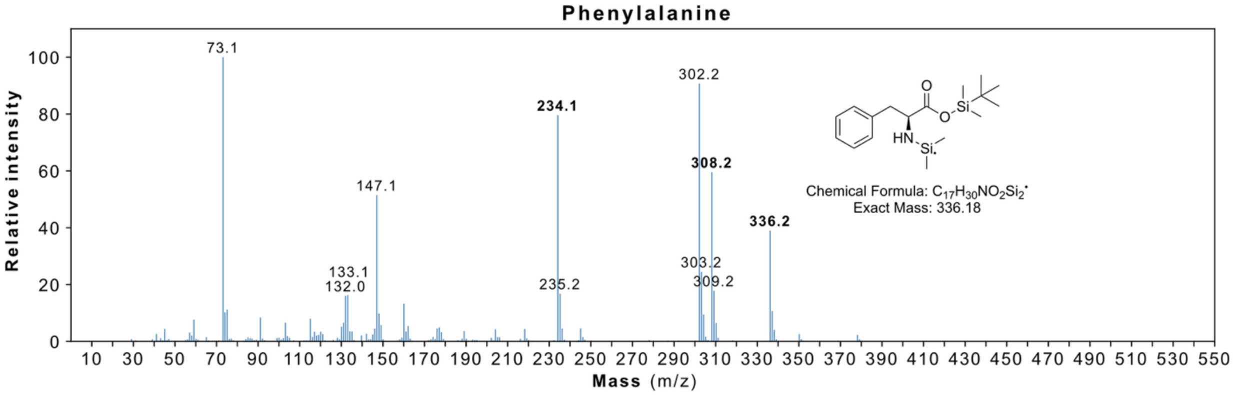

Figure S18. **Serine, 3tBDMS**. Full scan (*m*/*z* 10 - 550) mass fragmentation of a trisilylated serine derivative. The selected precursor ions are *m*/*z* **390.2** and **362.2**.

Molecular ion [M$^+$]: $C_{21}H_{49}NO_3Si_3$ (*m*/*z* 447.30)

[M$^+$] - *tert*-butyl (*t*B): $C_{17}H_{40}NO_3Si_3^{\bullet}$ (*m*/*z* 390.23)

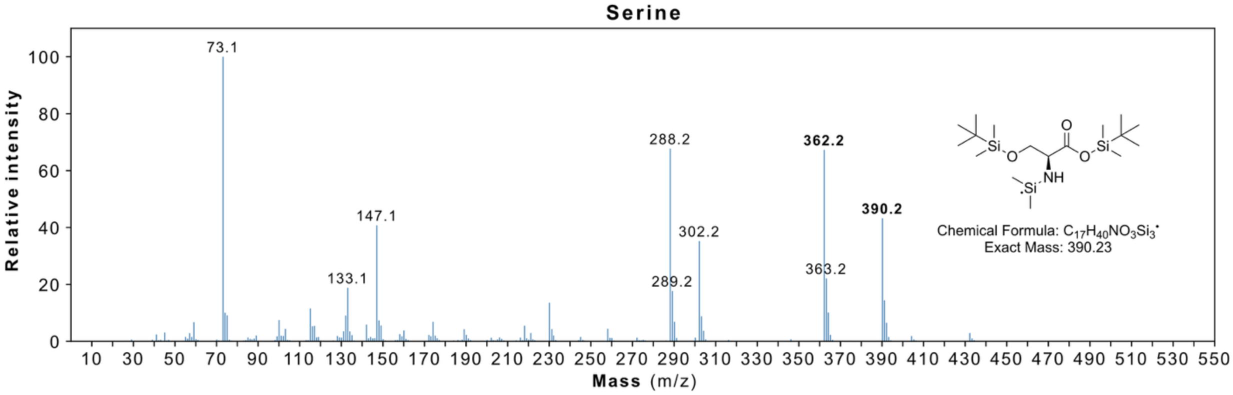

Figure S19. **Threonine, 3tBDMS**. Full scan (*m*/*z* 10 - 550) mass fragmentation of a trisilylated threonine derivative. The selected precursor ion is *m*/*z* **303.2**.

Molecular ion [M$^+$]: $C_{22}H_{51}NO_3Si_3$ (*m*/*z* 461.32)

[M$^+$] - *tert*-butyl (*t*B): $C_{18}H_{42}NO_3Si_3^{\bullet}$ (*m*/*z* 404.25)

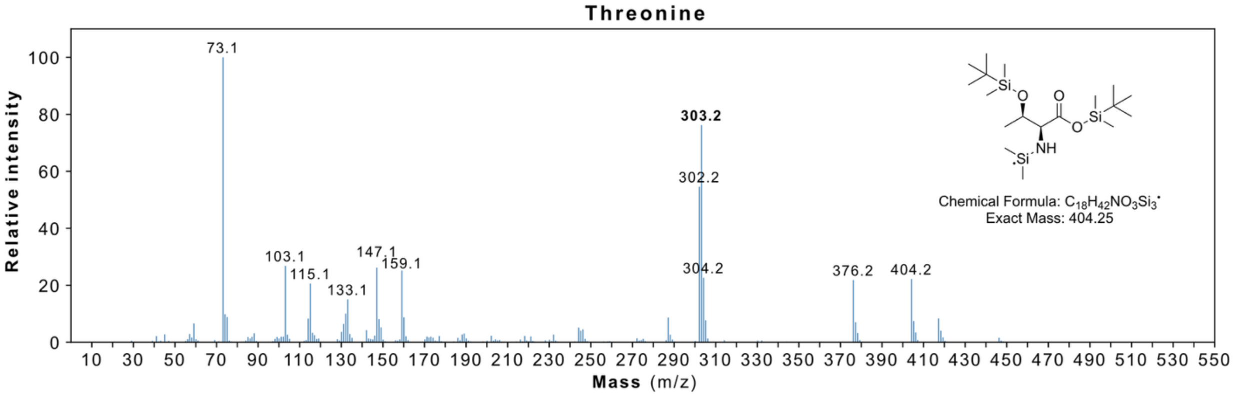

Figure S20. **Asparagine, 3tBDMS**. Full scan (*m*/*z* 10 - 550) mass fragmentation of a trisilylated asparagine derivative. The selected precursor ions are *m*/*z* **417.2** and **302.2**.

Molecular ion [M^+]: $C_{22}H_{50}N_2O_3Si_3$ (*m*/*z* 474.31)

[M^+] - *tert*-butyl (*t*B): $C_{18}H_{41}N_2O_3Si_3$ (*m*/*z* 417.24)

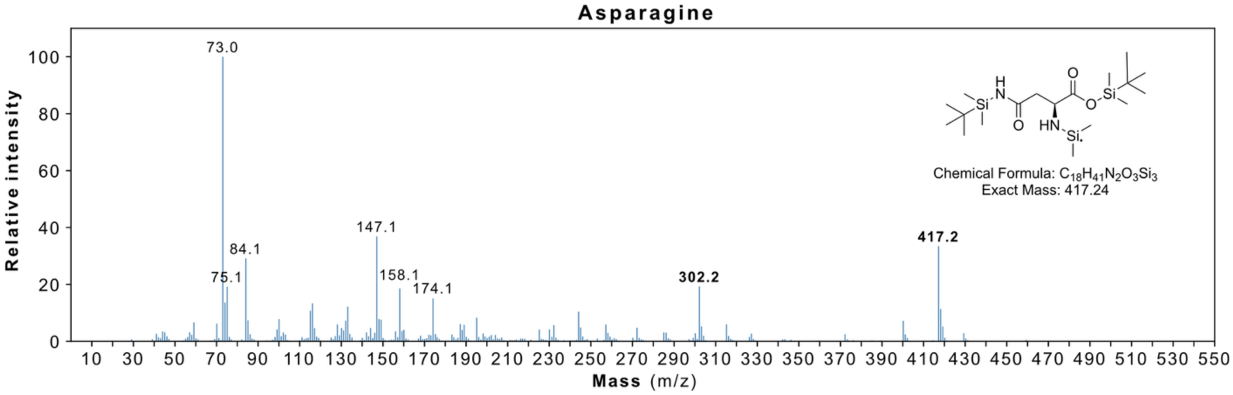

Figure S21. **Aspartic acid, 3tBDMS**. Full scan (*m*/*z* 10 - 550) mass fragmentation of a trisilylated aspartic acid derivative. The selected precursor ions are *m*/*z* **390.2** and **302.2**.

Molecular ion [M^+]: $C_{22}H_{49}NO_4Si_3$ (*m*/*z* 475.30)

[M^+] - *tert*-butyl (*t*B): $C_{18}H_{40}NO_4Si_3^{\bullet}$ (*m*/*z* 418.23)

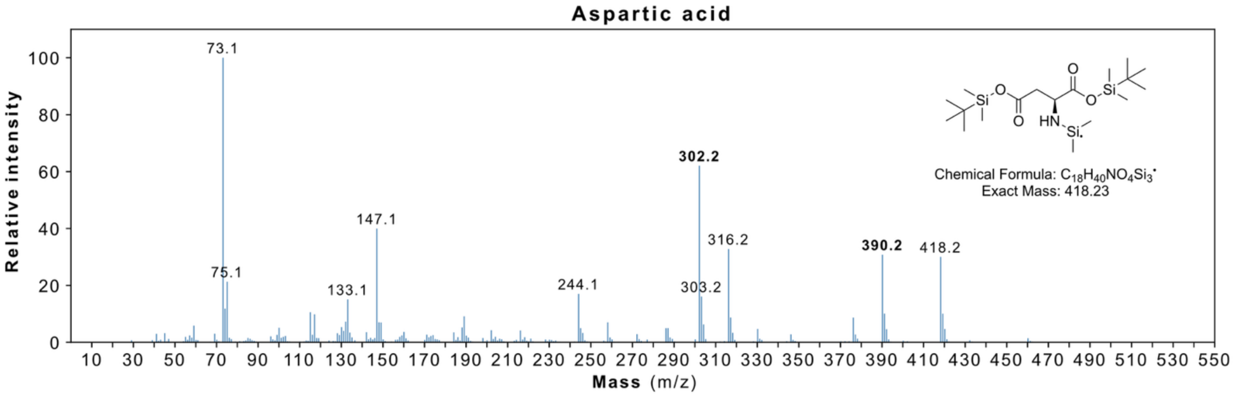

Figure S22. **Glutamine, 3tBDMS**. Full scan (*m*/*z* 10 - 550) mass fragmentation of a trisilylated glutamine derivative. The selected precursor ion is *m*/*z* **431.2**.

Molecular ion [M^+]: $C_{23}H_{52}N_2O_3Si_3$ (*m*/*z* 488.33)

[M^+] - *tert*-butyl (*t*B): $C_{19}H_{43}N_2O_3Si_3^{\bullet}$ (*m*/*z* 431.26)

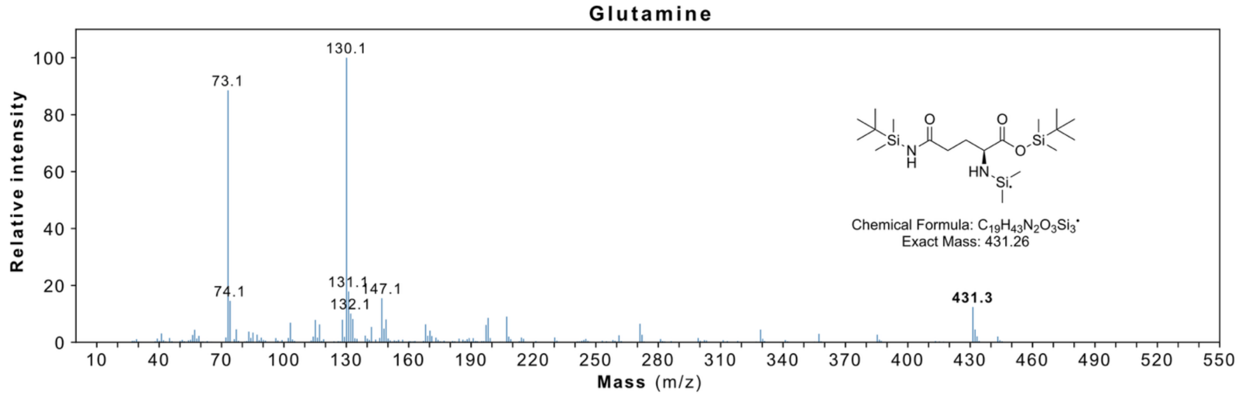

Figure S23. **Lysine, 3tBDMS**. Full scan (*m*/*z* 10 - 550) mass fragmentation of a trisilylated lysine derivative. The selected precursor ion is *m*/*z* **300.2**.

Molecular ion [M^+]: $C_{24}H_{56}N_2O_2Si_3$ (*m*/*z* 488.36)

[M^+] - *tert*-butyl (*t*B): $C_{20}H_{47}N_2O_2Si_3^{\bullet}$ (*m*/*z* 431.29)

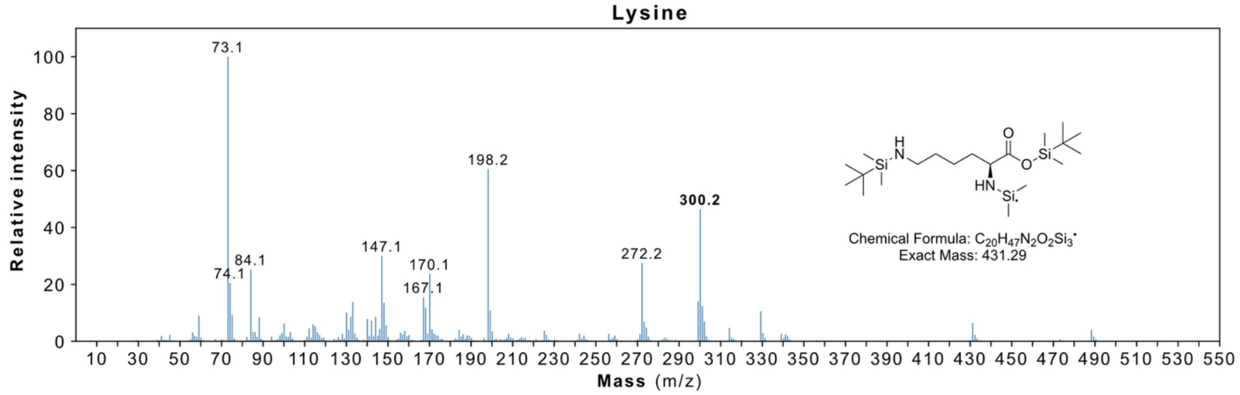

Figure S24. **Glutamic Acid, 3tBDMS**. Full scan (*m*/*z* 10 - 550) mass fragmentation of a trisilylated glutamic acid derivative. The selected precursor ions are *m*/*z* **432.3**, **330.2**, and **272.2**.

Molecular ion [M$^+$]: $C_{23}H_{51}NO_4Si_3$ (*m*/*z* 489.31)

[M$^+$] - *tert*-butyl (*t*B): $C_{19}H_{42}NO_4Si_3^{\bullet}$ (*m*/*z* 432.24)

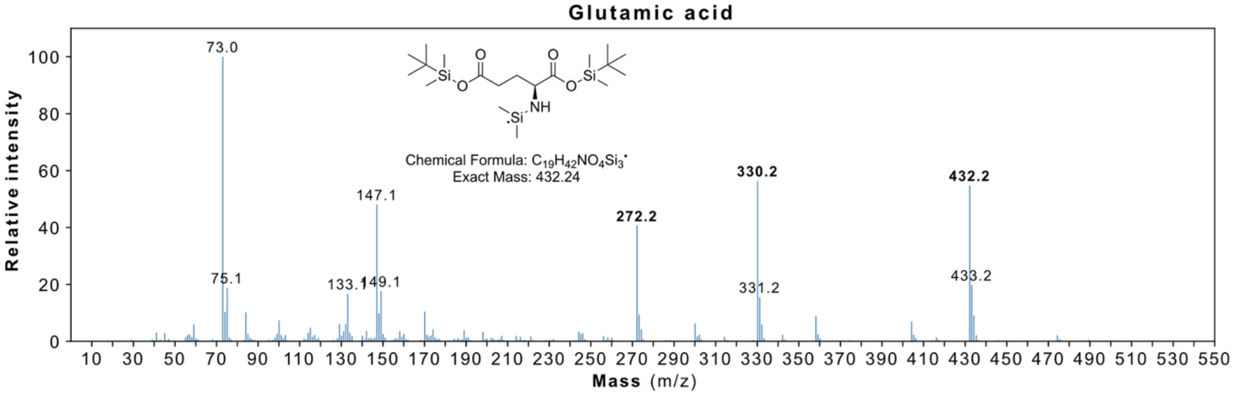

Figure S25. **Histidine, 3tBDMS**. Full scan (*m*/*z* 10 - 550) mass fragmentation of a trisilylated histidine derivative. The selected precursor ions are *m*/*z* **440.3** and **338.3**.

Molecular ion [M$^+$]: $C_{24}H_{51}N_3O_2Si_3$ (*m*/*z* 497.33)

[M$^+$] - *tert*-butyl (*t*B): $C_{20}H_{42}N_3O_2Si_3^{\bullet}$ (*m*/*z* 440.26)

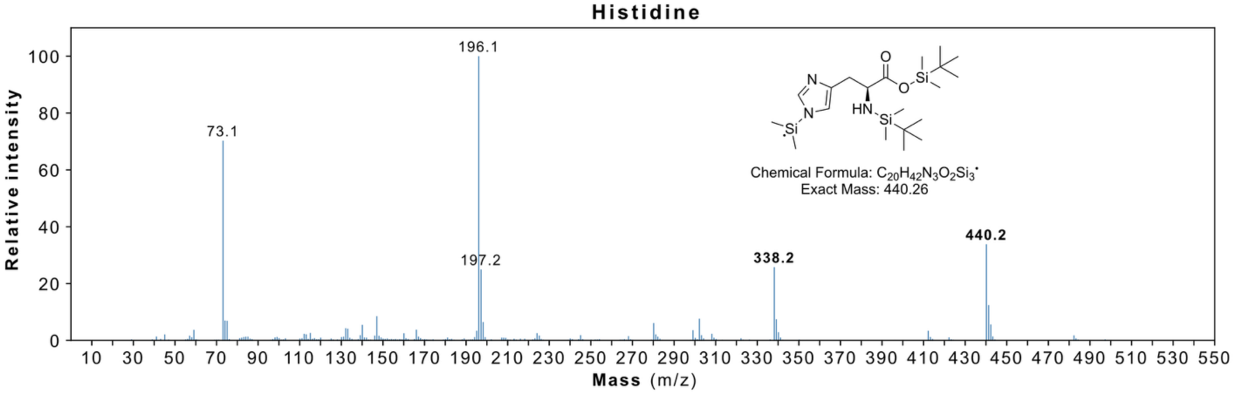

Figure S26. **Tyrosine, 3tBDMS**. Full scan (*m*/*z* 10 - 550) mass fragmentation of a trisilylated tyrosine derivative. The selected precursor ion is *m*/*z* **302.2**.

Molecular ion [M^+]: $C_{27}H_{53}NO_3Si_3$ (*m*/*z* 523.33)

[M^+] - *tert*-butyl (*t*B): $C_{23}H_{44}NO_3Si_3$ (*m*/*z* 466.26)

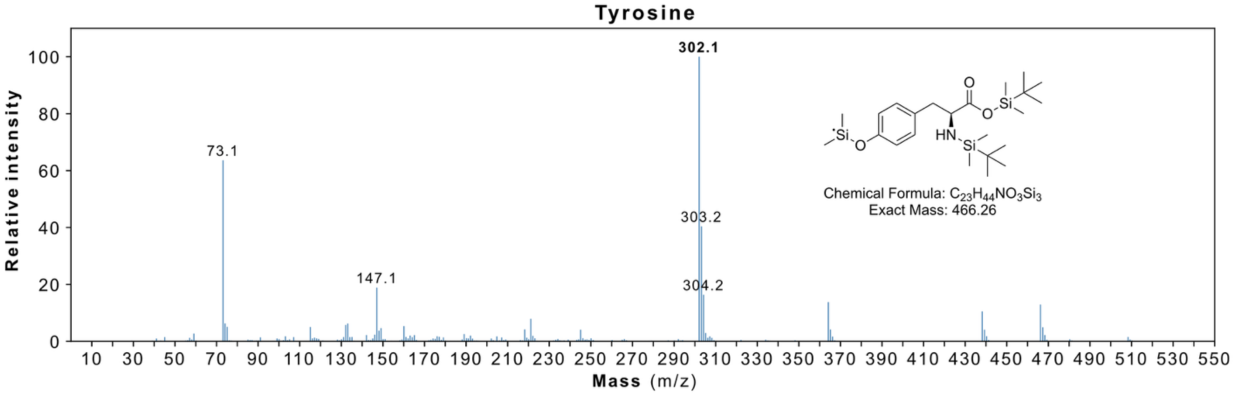

Figure S27. **Tryptophan, 3tBDMS**. Full scan (*m*/*z* 10 - 550) mass fragmentation of a trisilylated tryptophan derivative. The selected precursor ions are *m*/*z* **245.2** and **244.2**.

Molecular ion [M^+]: $C_{29}H_{54}N_2O_2Si_3$ (*m*/*z* 546.35)

[M^+] - *tert*-butyl (*t*B): $C_{25}H_{45}N_2O_2Si_3^{\bullet}$ (*m*/*z* 489.28)

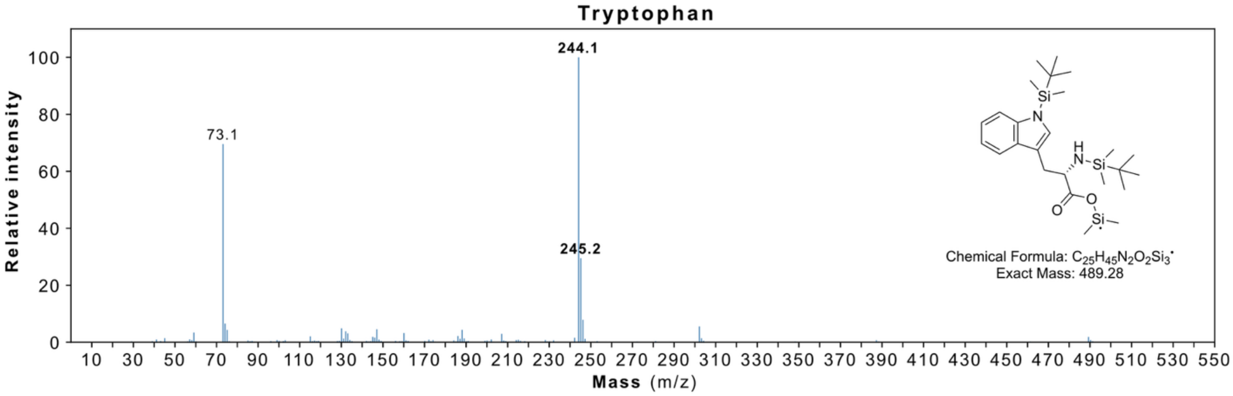

Figure S28. **Cystine, 4tBDMS** (Cysteine dimer). Full scan (*m*/*z* 10 - 550) mass fragmentation of a tetrasilylated cystine derivative. The selected precursor ion is *m*/*z* **348.2**.

Molecular ion [M$^+$]: $C_{30}H_{68}N_2O_4S_2Si_4$ (*m*/*z* 696.37)

[M$^+$] - *tert*-butyl (*t*B): $C_{26}H_{59}N_2O_4S_2Si_4^{\bullet}$ (*m*/*z* 639.30)

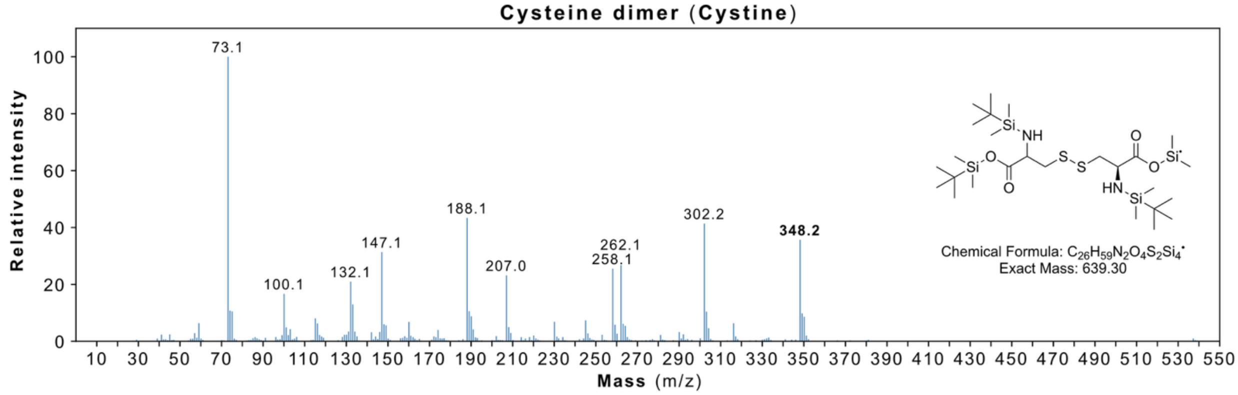

Figure S29. **Arginine, 4tBDMS**. Full scan (*m*/*z* 10 - 550) mass fragmentation of a tetrasilylated arginine derivative. The selected precursor ion is *m*/*z* **442.3**.

Molecular ion [M$^+$]: $C_{30}H_{70}N_4O_2Si_4$ (*m*/*z* 630.46)

[M$^+$] - *tert*-butyl (*t*B): $C_{26}H_{61}N_4O_2Si_4^{\bullet}$ (*m*/*z* 573.39)

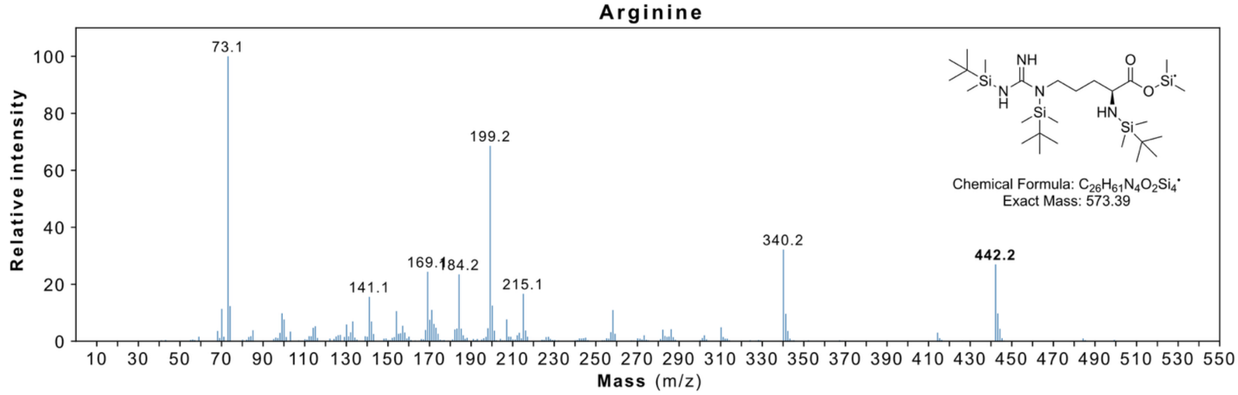

Nucleobase structures and mass spectra. Shown are full scan (*m/z* 10 - 550) fragmentation patterns, selected precursor ions (bolded), and structures from derivatized canonical nucleobases, xanthine, and hypoxanthine. Retention times and ion transitions are listed in **Table S3**

Figure S30. **Uracil, 2tBDMS**. Full scan (*m/z* 10 - 550) mass fragmentation of a bisilylated uracil derivative. The selected precursor ion is *m/z* **283.1**.

Molecular ion [M^+]: $C_{16}H_{32}N_2O_2Si_2$ (*m/z* 340.20)

[M^+] - *tert*-butyl (*t*B): $C_{12}H_{23}N_2O_2Si_2^{\bullet}$ (*m/z* 283.13)

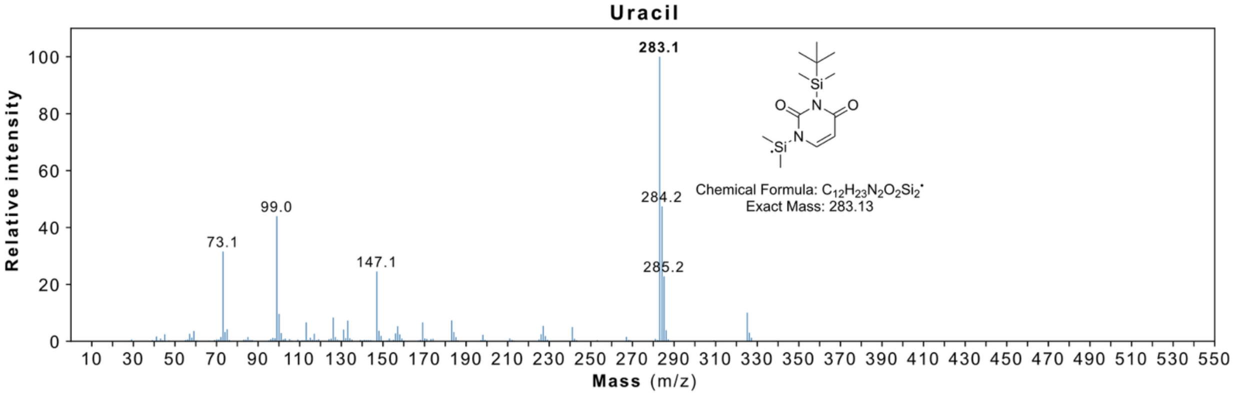

Figure S31. **Thymine, 2tBDMS**. Full scan (*m/z* 10 - 550) mass fragmentation of a bisilylated thymine derivative. The selected precursor ion is *m/z* **297.1**.

Molecular ion [M^+]: $C_{17}H_{34}N_2O_2Si_2$ (*m/z* 354.22)

[M^+] - *tert*-butyl (*t*B): $C_{13}H_{25}N_2O_2Si_2^{\bullet}$ (*m/z* 297.15)

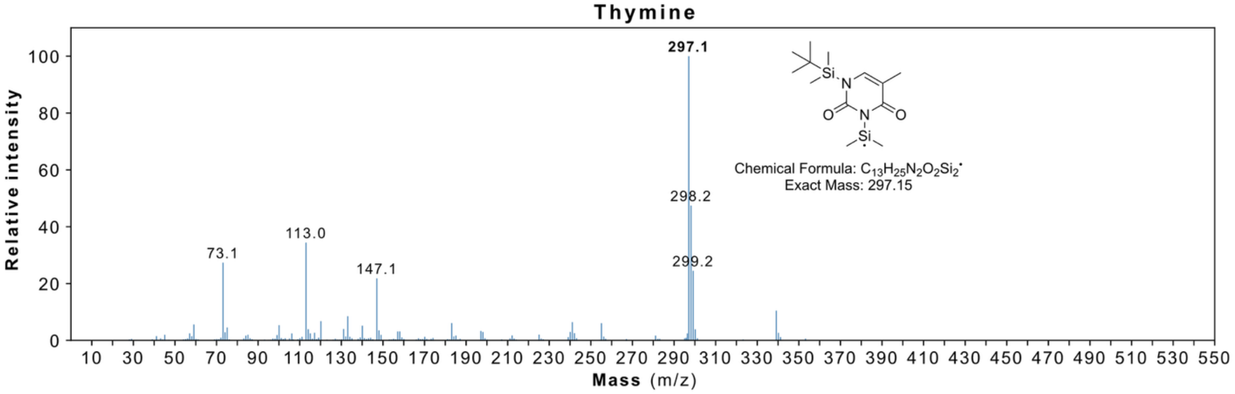

Figure S32. **Cytosine, 2tBDMS**. Full scan (*m*/*z* 10 - 550) mass fragmentation of a bisilylated cytosine derivative. The selected precursor ions are *m*/*z* **283.2** and **282.1**.

Molecular ion [M+]: $C_{16}H_{33}N_3OSi_2$ (*m*/*z* 339.22)

[M+] - *tert*-butyl (*t*B): $C_{12}H_{24}N_3OSi_2^{\bullet}$ (*m*/*z* 282.15)

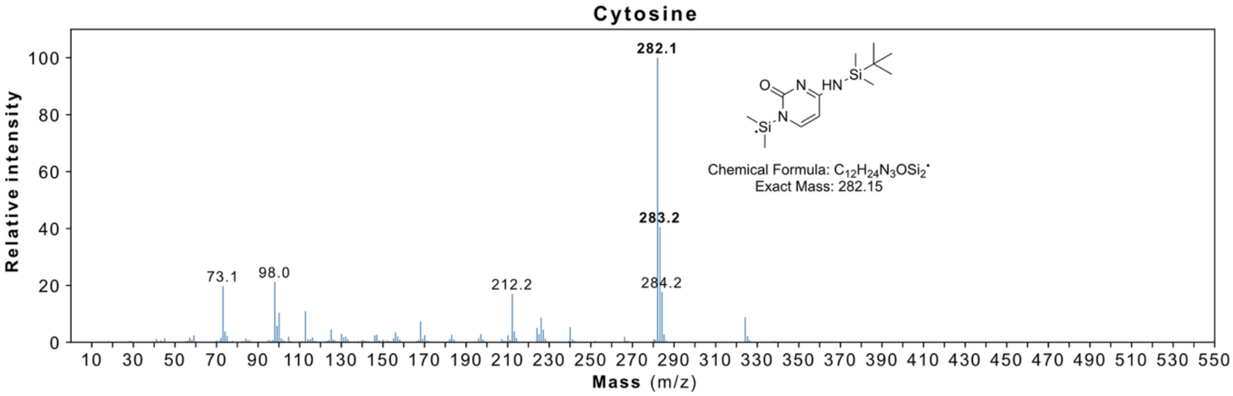

Figure S33. **Adenine, 2tBDMS**. Full scan (*m*/*z* 10 - 550) mass fragmentation of a bisilylated adenine derivative. The selected precursor ions are *m*/*z* **307.2**, **306.2**, and **192.1**.

Molecular ion [M+]: $C_{17}H_{33}N_5Si_2$ (*m*/*z* 363.23)

[M+] - *tert*-butyl (*t*B): $C_{13}H_{24}N_5Si_2^{\bullet}$ (*m*/*z* 306.16)

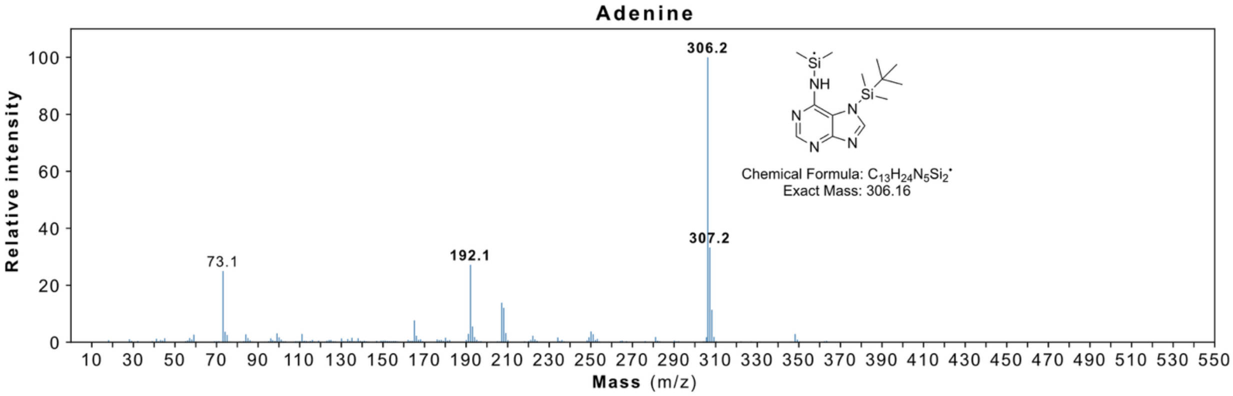

Figure S34. **Hypoxanthine, 2tBDMS**. Full scan (*m*/*z* 10 - 550) mass fragmentation of a bisilylated hypoxanthine derivative. The selected precursor ions are *m*/*z* **307.2** and **193.1**.

Molecular ion [M^+]: $C_{17}H_{32}N_4OSi_2$ (*m*/*z* 364.21)

[M^+] - *tert*-butyl (*t*B): $C_{13}H_{23}N_4OSi_2^{\bullet}$ (*m*/*z* 307.14)

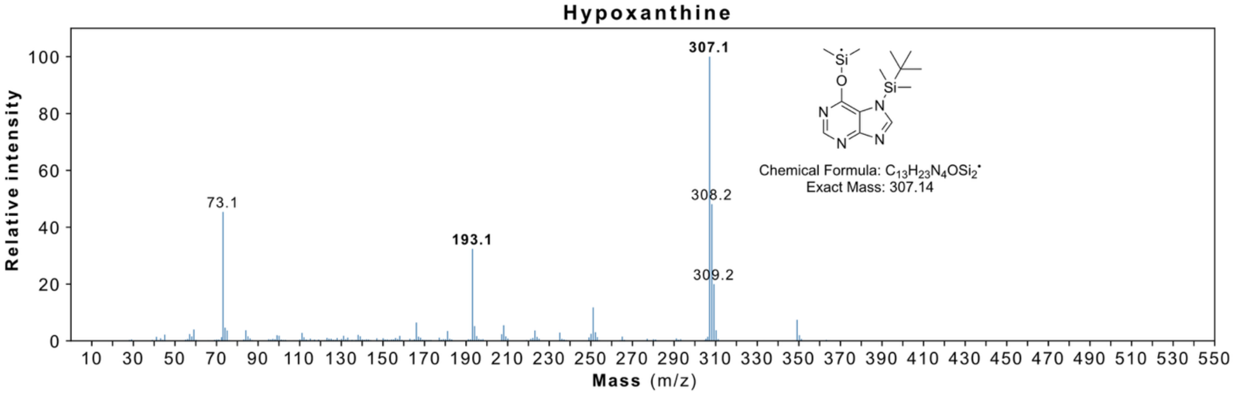

Figure S35. **Guanine, 3tBDMS**. Full scan (*m*/*z* 10 - 550) mass fragmentation of a trisilylated guanine derivative. The selected precursor ion is *m*/*z* **436.3**.

Molecular ion [M^+]: $C_{23}H_{47}N_5OSi_3$ (*m*/*z* 493.31)

[M^+] - *tert*-butyl (*t*B): $C_{19}H_{38}N_5OSi_3^{\bullet}$ (*m*/*z* 436.24)

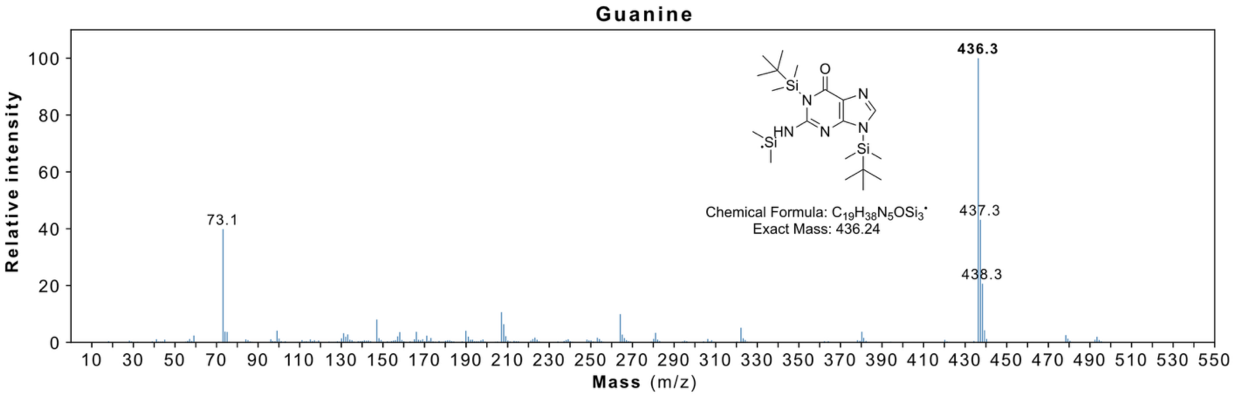

Figure S36. **Xanthine, 3tBDMS**. Full scan (*m*/*z* 10 - 550) mass fragmentation of a trisilylated xanthine derivative. The selected precursor ion is *m*/*z* **437.2**.

Molecular ion [M^+]: $C_{23}H_{46}N_4O_2Si_3$ (*m*/*z* 494.29)

[M^+] - *tert*-butyl (*t*B): $C_{19}H_{37}N_4O_2Si_3^{\bullet}$ (*m*/*z* 437.22)

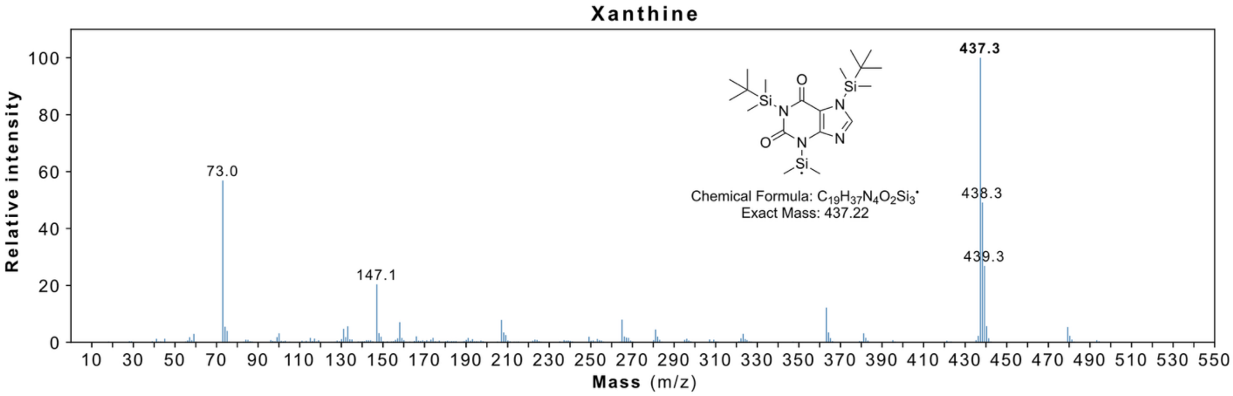

Supplement: Supplementary file 1 — Appendix 01 (PDF) [file pnas.2512461122.sapp.pdf]
